# Mesoscale visualization of three-dimensional microvascular architecture and immunocyte distribution in intact mouse liver lobes

**DOI:** 10.7150/thno.71718

**Published:** 2022-07-11

**Authors:** Zheng Liu, Mengli Xu, Songlin Huang, Qi Pan, Chong Liu, Fanxin Zeng, Zhan Fan, Yafang Lu, Jialu Wang, Jinxin Liu, Xinlin Li, Qingming Luo, Zhihong Zhang

**Affiliations:** 1 Britton Chance Center and MoE Key Laboratory for Biomedical Photonics, Wuhan National Laboratory for Optoelectronics-Huazhong University of Science and Technology, Wuhan, Hubei 430074, China.; 2 School of Biomedical Engineering, Hainan University, Haikou, Hainan 570228, China.

**Keywords:** liver-CUBIC, liver vessel, immune cell, hepatic lobule, liver metastases

## Abstract

**Rational:** The complex vascular architecture and diverse immune cells of the liver are critical for maintaining liver homeostasis. However, quantification of the network of liver vasculature and immunocytes at different scales from a single hepatic lobule to an intact liver lobe remains challenging.

**Methods:** Here, we developed a fast and fluorescence-preserving transparency method, denoted liver-CUBIC, for systematic and integrated analysis of the microcirculation and the three-dimensional distribution of dendritic cells (DCs)/macrophages in intact liver lobes.

**Results:** Whole-mount imaging at mesoscale revealed that the hepatic classical lobule preferred the oblate ellipsoid morphology in the mouse liver and exhibited hepatic sinusoids with heterogeneous arrangement and intricate loop structure. Liver fibrosis not only induces sinusoidal density increase but also promotes sinusoidal arrangement with increased sinusoidal branch and loop structure. Meanwhile, we found that CD11c^+^ DCs followed a lognormal distribution in the hepatic lobules, skewing toward lobular boundary in steady state. CCl_4_-induced chronic liver injury promoted CD11c^+^ DC rearrangement at the lobular border before the formation of liver fibrosis. Furthermore, through whole-mount imaging of tumor-immune cell-vascular crosstalk in intact lobes based on liver-CUBIC, we characterized an accumulation of CX3CR1^+^CCR2^+^F4/80^+^ macrophages at metastatic foci in early colorectal liver metastases. Importantly, colorectal cells secrete CCL2 to mobilize CX3CR1^+^CCR2^+^F4/80^+^ macrophages to accumulate at liver micrometastases, and the interruption of CCL2-induced macrophage accumulation inhibits early colonization of metastasis in the liver.

**Conclusions:** Our investigation of the sinusoidal network and DC/macrophage arrangements through the liver-CUBIC approach and whole-mount imaging provide a powerful platform for understanding hepatic circulatory properties and immune surveillance in the liver.

## Introduction

The liver is the most important metabolic and detoxification organ in the body and has a high incidence of refractory disease, which is closely related to its unique anatomical structure, complex vascular network, and immune tolerance status 1. Changes in blood vessels and imbalance of the immune environment in the liver occur in most hepatic diseases, such as hepatitis, hepatic fibrosis, and liver metastasis 2-5. Thus, an understanding of the fine vascular structure of the liver and the distribution of immune cells in the intact structural unit is of great significance for understanding the function of the liver.

The liver has a complex cross-scale vasculature, and defining the basic structure and functional unit of the liver has been challenging since the hepatic lobules were first described in 1665 1, 6. Although the macrovascular structure of the liver, including the hepatic artery (HA), portal vein (PV), and hepatic vein (HV), is well understood, the features of the liver microcirculation remain unclear 7. The hexagonal lobule theory established by Kiernan in 1833 describes a widely accepted standard microstructure for the liver at the microanatomical level 8. In this model, the central vein (CV) is located in the middle of the hepatic classic lobules and is the terminal branch of the HV. The portal triads (PTs) consist of the PV, HA and bile duct and are distributed at the boundary between two adjacent hepatic classic lobules. However, this hexagonal lobule is a simplification of the complex three-dimensional (3D) microvascular architecture of the liver 9. In addition, according to the distribution characteristics of hepatic vascular branches, the liver organization units could also be defined as portal lobules in which the portal tract is located at the center, and the CV is located at each corner 10. A more detailed analysis of the 3D anatomical structure of the hepatic circulation at different scales is meaningful for understanding its function, vascular modeling, hemodynamics, and role in various liver pathologies 11-13.

The liver is also a critical organ for immune surveillance throughout life. Dendritic cells (DCs) and macrophages are strategically positioned in the liver and constitute a tight defense network that resists the invasion of pathogens or clears apoptotic liver cells 14, 15. The function of DCs/macrophages is closely related to their spatial location and environment. For example, the CX3CR1^+^ DC subset distributed under the mesothelium plays an important role in antigen processing and presentation in the liver 16; CX3CR1^+^CD207^+^ macrophages located in the capsular region defend against pathogen invasion from the peritoneum 17; Kupffer cells distributed in the sinusoidal lumen clear invading pathogens from the bloodstream 18. Recently, an increasing number of studies have shown that DCs and macrophages play a critical role in the occurrence and development of liver diseases, such as liver metastasis 3, alcoholic liver disease 19, nonalcoholic steatohepatitis 20, and viral hepatitis 21. Thus, uncovering the arrangement of DCs/macrophages in an intact liver lobe at single-cell resolution will provide a better understanding of the function and heterogeneity of DCs/macrophages in liver physiology and pathology.

To date, it is still challenging to study the network of liver vessels and immune cells at the mesoscale, ranging from liver immune cells at the micron level to liver vessels at the millimeter level. Imaging data based on a small-scale slice of the liver lack overall spatial location information. Although computed tomography (CT) and magnetic resonance imaging (MRI) techniques have accelerated research on the liver structure 22, 23, simultaneously obtaining liver immune cells and hepatic sinusoid structure information from intact lobes at single-cell resolution is difficult. Due to the rough understanding of the structure of the liver, the theoretical model based on neatly arranged hexagonal columns is still used in the description of the basic structural unit of the hepatic lobule 24. Previously, our large field of view photoacoustic microscopy results showed that the hepatic lobules show a crisscross pattern and are very elaborate in mice 25. Therefore, a complete three-dimensional structural map of the liver at the mesoscopic scale is needed to better understand the arrangement of the hepatic lobules and the connection of immune cells with the vascular system.

Optical microscopic imaging combined with optical transparency is a well-received method that has frequently been used to obtain information on the distribution of individual cells in a whole organ 26-29. Here, we developed an advanced clearing technology based on CUBIC 30, 31 by introducing lipase to pretreat the liver tissue and regulate the pH of the clearing reagents, named liver-CUBIC. We found that liver-CUBIC achieves good transparency of the liver with a shorter time for liver clearing and better fluorescence preservation than the CUBIC approach. Through confocal imaging of the cleared liver lobes, we obtained 3D structural information for hepatic lobules, extracted the topology of the hepatic sinusoid network, and uncovered the spatial distribution of DCs/macrophages in each hepatic lobule of normal or diseased mice.

## Methods

### Animals

We used the following animals in this study. CX3CR1-EGFP (Stock No. 005582), CD11c-YFP (Stock No. 008829), mTmG (Stock No. 007676), LysM-Cre (Stock No. 004781), and LSL-RFP (Stock No. 007909) mice were obtained from the Jackson Laboratory (Bar Harbor, ME, USA). LysM-Cre mice were crossed with LSL-RFP mice to produce LysM-RFP mice, and CX3CR1-EGFP mice were crossed with mTmG mice to produce CX3CR1-EGFP/mTmG mice. The mice (aged 4-14 weeks) used in this paper were bred and maintained in a specific pathogen-free mouse room under a 12-h light/12-h dark cycle. All mouse experiments were in accordance with the Experimental Animal Management Ordinance of Hubei Province, P. R. China, and the guidelines of Huazhong University of Science and Technology and were approved by the Institutional Animal Ethics Committee of Huazhong University of Science and Technology (IACUC Number: 844).

### Cell lines

MC38 cells were transfected with the PB transposon system (a gift from Dr. Xiaohui Wu, Fudan University, Shanghai, China), which contained the sequence encoding mCherry, to generate the mCherry-MC38 tumor cell line. The MC38 cell line was obtained from Professor Tian (University of Science and Technology of China, Anhui, China). The cells were cultured in DMEM (HyClone, SH30022) containing 10% FBS (Gibco, 10270106) and 100 U/mL penicillin-streptomycin (Gibco, 15070063) in an incubator (Thermo, USA) in an atmosphere with 5% CO_2_ at 37 °C.

### Liver metastasis model

To perform liver metastasis, CX3CR1^gfp/+^ heterozygous mice and CX3CR1-deficient mice were anesthetized with a mixture of 10 mg/kg xylazine and 100 mg/kg ketamine hydrochloride (Sigma, USA), and 2 × 10^5^ tumor cells in 100 μL of PBS were injected into the hemispleen (the part is closest to the liver) within 2-3 min. Five min later, the syringe was removed, and the incision was closed. For CCL2 blockade, CX3CR1^gfp/+^ heterozygous mice received an intravenous tail injection of 200 μg of anti-CCL2 antibodies (Armenian Hamster IgG, κ, clone 2H5, catalog BE0185) or IgG isotype (Armenian hamster IgG, catalog BE0091) before tumor cell inoculation and on the second day following tumor cell inoculation. The body temperature of the mice was maintained at 37 °C with a warmer plate (RWD, Shenzhen, China) during the experiment. The mice were perfused on day 3 for the following clearing experiment.

### Carbon tetrachloride (CCl_4_) induces chronic liver injury and fibrosis

Liver fibrosis was induced in adult C57 and CD11c-venus mice through an intraperitoneal injection of a solution of 25% CCl_4_ in corn oil twice weekly, and the dosage was 40 μL per 20 g mouse. To extract the DC rearrangement in chronic liver injury and fibrosis, liver lobes from CD11c-venus mice were harvested for clearing 3-week or 6-week periods of CCl_4_ injections. To extract the sinusoidal microcirculation in liver fibrosis, liver lobes from C57 mice were harvested for clearing after a 6-week period of CCl_4_ injections.

### CUBIC and liver-CUBIC protocols

The CUBIC and liver-CUBIC clearing protocols are shown in the supplementary data. In brief, anesthetized mice were first transcardially perfused with PBS, and the liver lobe volume was then measured using a drainage strategy. The ratio of the cleared lobe volume to the original lobe volume was defined as the change in the liver lobe size after clearing. The linear size change value was quantified as the cube root of the change in the volume.

### Immunofluorescence staining

The liver lobes were fixed with 4% paraformaldehyde (PFA) for 1 h at 4 °C and cut into 50-μm sections with a VT1200 vibratome (Leica, Germany). These liver sections were washed three times in PBS (1 min per wash) and then blocked with PBS/1% BSA/0.2% Triton X-100 for 2 h at 4 °C. After blocking, the liver sections were separately stained with PE anti-mouse MHC-II (BioLegend, clone: M5/114.15.2), AF594 anti-mouse F4/80 (BioLegend, clone: BM8), PE anti-mouse Ly6C (BioLegend, clone: HK1.4) and primary rabbit anti-mouse CCR2 (Abcam, clone: EPR20844) antibodies overnight at 4 °C. The sections were then washed three times in PBS (1 min per wash). For rabbit anti-mouse CCR2 staining, the sections were washed four times in PBS/2% BSA/0.2% Triton X-100 (1 h per time) at room temperature, and the sections were further stained with the secondary antibody AF647 goat anti-rabbit IgG (Abcam, cat: ab150079) overnight at 4 °C and washed again in PBS/2% BSA/0.2% Triton X-100. The details of the antibodies are shown in Supplementary Table 2. All antibodies were diluted in PBS/1% BSA/0.2% Triton X-100. All liver sections were imaged with an LSM 710 confocal microscope (Zeiss, Germany). The 3D immunofluorescence staining of the liver was performed referring CUBIC-HistoVIsion 32 with some modification, and the details are shown in the **Supplementary Methods**.

**Please refer to the supplementary data for additional methods.**

## Results

### Development of a liver-CUBIC method for liver clearing

**Figure 1:**
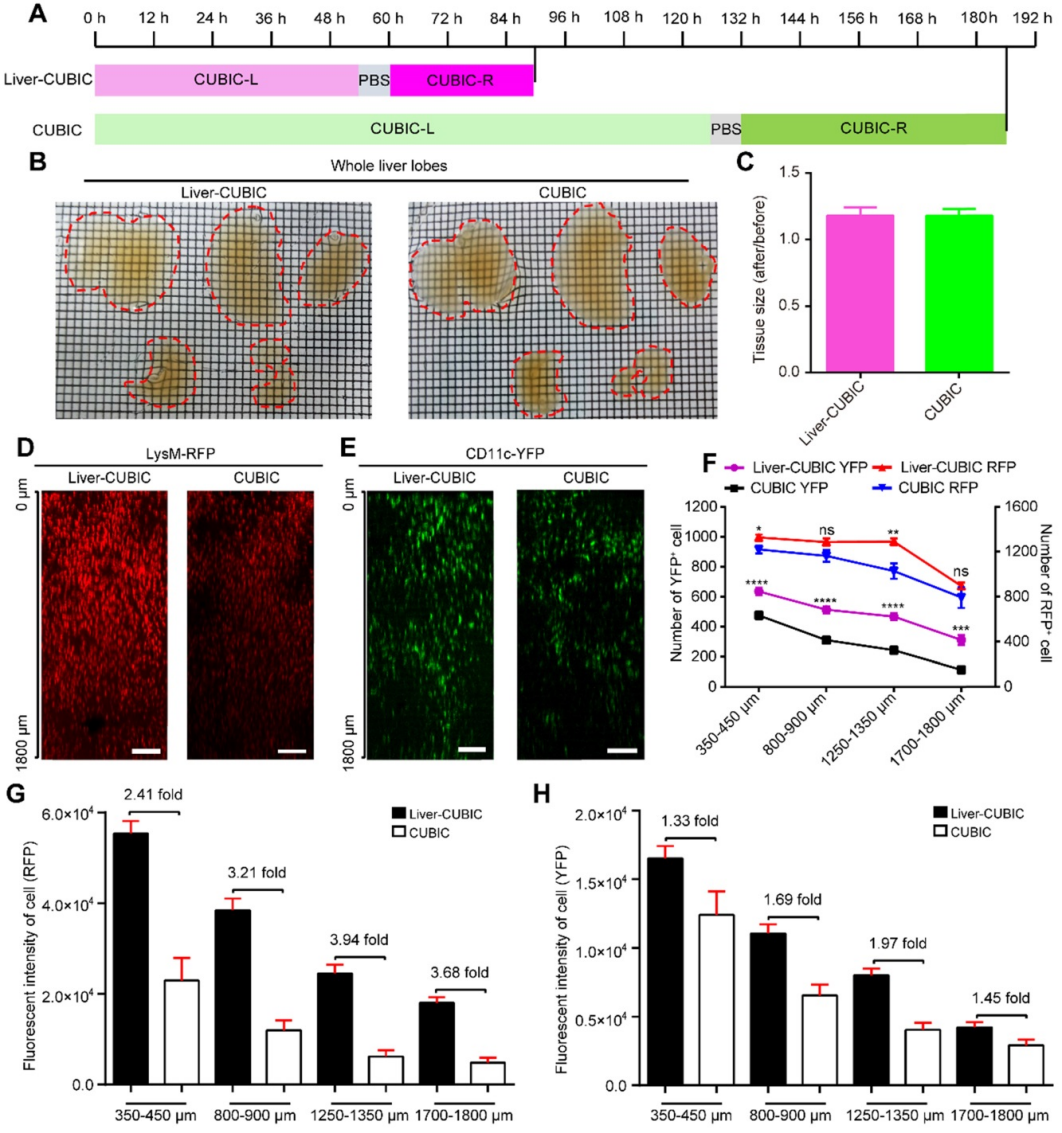
** Development of liver-CUBIC. (A)** Clearing time and protocol of liver-CUBIC and CUBIC for the whole liver. **(B)** Bright-field images of the intact liver from CX3CR1-EGFP mice (aged 8-9 weeks, male) cleared with liver-CUBIC (left) and CUBIC (right). The scale of the grid is 1.6 mm × 1.6 mm. **(C)** Quantification of the tissue size change after clearing (n = 4 samples for each method). Error bars denote the SEMs. **(D-E)** Comparison of the imaging depth of RFP and YFP for LysM-RFP and CD11c-YFP mouse livers with different methods. Confocal imaging (10×, NA = 0.45) of 1.8-mm-thick liver sections. Z-steps, 5 µm. The y-direction was a 50-µm maximum intensity projection. Scale bar: 200 µm. **(F)** The 1.8-mm-thick liver was selected from four different depths for analysis. Under different transparency conditions, the cell numbers in each depth layer were automatically detected based on RFP and YFP fluorescence (n = 9 measurements per group). Error bars denote the SEMs. **(G-H)** Quantitative analysis of fluorescence intensity under different liver depths and with different transparency methods. Each value represents the average fluorescence intensity of total RFP^+^ or YFP^+^ cells at each depth. The data were collected from 9 image regions per group. Error bars denote the SEMs. **(B-H)** All the data were obtained from three independent repeated experiments; ns, not significant, *P < 0.1, **P < 0.01, ***P < 0.001, ****P < 0.0001, Mann-Whitney U test.

The CUBIC method is a powerful approach that has achieved good transparency of the mouse liver 30, 33. However, the low retention of fluorescence signals after liver clearing restricts the application of CUBIC in the acquisition of fine vascular structures and immunocyte distribution in intact liver lobes. Seeking a fast and transparent method to improve fluorescence preservation in CUBIC is necessary. Recently, Mengjie Lai *et al*. achieved good transparency of liver slices by adding a defatting step during SDS clearing 34. However, this approach takes 12 days to clear 1-mm-thick slices of liver. Inspired by these studies, we wondered whether a defatting step could speed up the transparency time of CUBIC. In this study, a defatting step involving perfusion with a saturated lipase solution was added to pre-defat the mouse tissues after the mice had been transcardially perfused with PFA. The inclusion of this step reduced the immersion time from 7.75 days (the time needed in the CUBIC approach) to 3.75 days (Figure 1A). The resulting approach was termed liver-CUBIC.

Bright-field imaging indicated that liver-CUBIC achieved good transparency of the liver (Figure 1B). No significant difference in the expansion rates of liver samples was found between liver-CUBIC and CUBIC (Figure 1C). We then tested the compatibility of fluorescent proteins in the liver-CUBIC method using a CD11c-YFP-labeled mouse strain (mainly CD11c^+^ DCs) and a LysM-RFP-labeled mouse strain (mainly LysM^+^ macrophages) 35. The data showed that the liver-CUBIC method achieves a brighter and deeper fluorescent signal than the CUBIC method when imaging liver lobes (Figure 1D-E). A quantitative evaluation of the signal retention ability for fluorescent proteins confirmed that the number of detectable YFP cells at different depths in the liver-CUBIC-treated lobes was significantly greater than that in the CUBIC group (Figure 1F-H, Supplementary Movies 1-2). The number of YFP cells detected in the liver-CUBIC group was 1.3-fold higher than that in the CUBIC group at 350 μm to 450 μm and 2.7-fold higher than that in the CUBIC group at 1700 μm to 1800 μm (Figure 1F). Furthermore, the fluorescence intensity per YFP-positive cell in the liver-CUBIC group was 1.3-2.0-fold higher than that in the CUBIC group at different depths (Figure 1H, S1). Although little difference in the number of detected RFP cells between the liver-CUBIC and CUBIC groups was found, the fluorescence intensity per RFP-positive cell in the liver-CUBIC group was 2.4-3.9-fold higher than that in the CUBIC group at different depths (Figure 1F, G, Figure S1).

Because GFP fluorescence is sensitive to pH and temperature 36, the liver samples were then maintained at 4 °C and pH 9.5 during refractive index matching, resulting in enhanced retention of GFP fluorescence in the liver samples (Figure S2). The liver-CUBIC approach is compatible with the clearing of other organs, such as the kidney, spleen and thymus, and is also compatible with 3D immunostaining of the liver lobe (Figures S3 and S4). The iDISCO approach also reportedly achieves imaging of the hepatic sympathetic nerve in the intact liver lobe 37, 38. Here, we further compared the liver-CUBIC approach with the iDISCO approach for endogenous fluorescence and exogenous fluorescence (e.g., antibody staining) imaging. As shown in Figure S5, the liver-CUBIC approach was found to be superior in the maintenance of endogenous fluorescence, whereas the iDISCO approach achieved a better fluorescence signal in anti-TH antibody labeling.

Thus, the established liver-CUBIC method not only shortened the clearing time for the liver but also preserved better fluorescence signals than CUBIC, which indicated that liver-CUBIC has more advantages and is able to acquire higher-quality cellular information and deeper fluorescence signals than CUBIC in liver clearing.

### Segmentation and visualization of the vascular structure and hepatic lobules in an intact liver lobe

**Figure 2:**
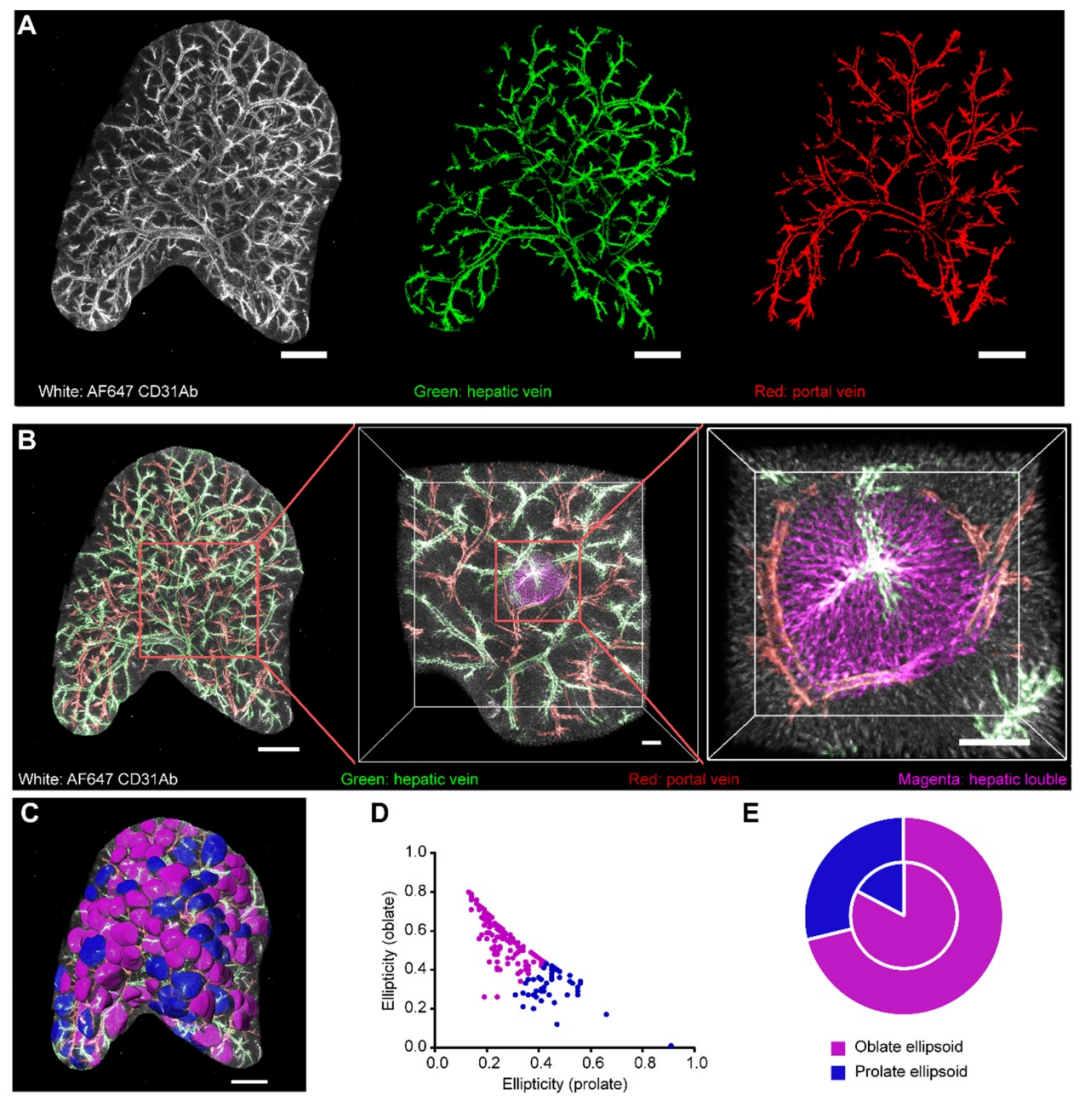
** 3D confocal imaging of intact liver lobes treated with liver-CUBIC.** Liver vasculature labeled with AF647-CD31Ab (white). **(A)** Vascular imaging and vascular segmentation of an intact liver lobe to distinguish the HV (green) and PV (red). Scale bar: 1000 µm. Z-steps, 26.3 µm. **(B)** Multiscale visualization of the fine vascular structure and a segmented hepatic lobule in an intact liver lobe. The HV is shown in green, the PV is shown in red, and the hepatic lobules are shown in magenta. The scale bar of the intact liver lobe is 1000 µm. The details in the red-boxed regions are shown on the right. Scale bar: 200 µm. **(C)** The hepatic lobules from (b) were segmented using “Imaris” software and labeled in false color. The hepatic lobules marked in magenta are oblate ellipsoids, and the hepatic lobules shown in blue are prolate ellipsoids. Scale bar: 1000 µm. **(D)** Quantification of the hepatic lobule ellipticity. The red points represent hepatic lobules with an oblate ellipsoid shape, and the blue points represent hepatic lobules with a prolate ellipsoid shape. **(E)** The pie chart shows the shape-based distribution of the hepatic lobules on the surface (outer circle) and interior (inner circle) of the liver lobe. The data in D-E were obtained from three independent repeated experiments. The thickness of the projections is 2.68 mm.

Having developed the liver-CUBIC method for liver clearing, we first acquired the vascular structure in intact liver lobes through systemic antibody injection of Alexa Fluor 647 (AF647)-CD31Ab (BioLegend, clone: MEC13.3). Based on the existing vascular architecture of HV, PV, HA, and CV obtained by micro-computed tomography (CT) 23, we distinguished HV from the AF647-CD31Ab-labeled vasculature through retrograde tracking of the CV in the liver (Figure 2A). As the PVs and HA were parallel to each other and the artery was thinner than the HV, we successfully segmented the HV (containing its branch of the CV) and PV (containing its branch of the interlobular vein) from the AF647-CD31Ab-labeled vasculature (Figure 2A, Figure S6, Supplementary movie 3). The 3D structure of the classical lobule was then segmented according to the direction of the liver sinusoids in each layer along the Z-axis, referring to the position of the interlobular vein and CV (Figure 2B-C, Supplementary movie 4). Analysis of the spherical rate and ellipticity of the 3D morphology of the classical lobule revealed that 72.58% of the lobular shape tended to be oblate ellipsoids, and 27.42% tended to be prolate ellipsoids (Figure 2D). Further analysis of the relationship between the morphology and location of the classical lobule on the surface of the liver lobes showed that 71% and 29% of the classical lobules tended to be oblate ellipsoids and prolate ellipsoids, respectively (Figure 2E). Interestingly, the ratio of oblate ellipsoids to prolate ellipsoids was higher in the interior of the liver lobes: 83% of the classical lobules tended to be oblate ellipsoids, and 17% of the classical lobules tended to be prolate ellipsoids.

Collectively, our imaging data of the intact liver lobes clearly revealed the shape and spatial location of the hepatic lobules of mice, which were not hexagonal columns as is generally considered, and the overall morphology of the hepatic lobules tended to be oblate ellipsoids, particularly in the interior of the liver lobes.

### Microcirculation of the sinusoidal system in intact hepatic lobules

**Figure 3:**
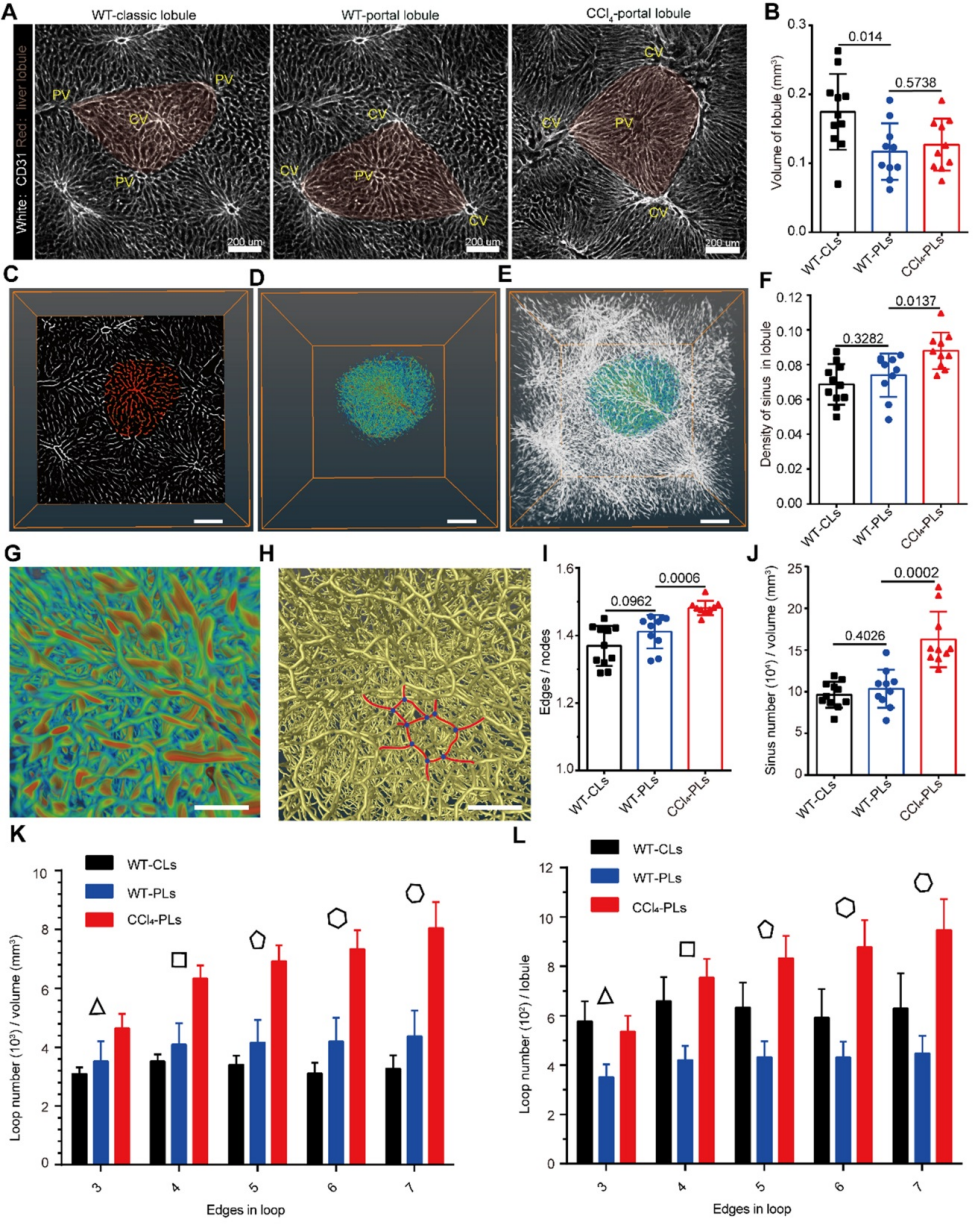
** Microcirculation and topology of the hepatic sinusoid system. (A-E)** Representative data of sinusoidal structures in normal classical lobules (WT-CLs), normal portal lobules (WT-PLs) and fibrotic portal lobules (CCl_4_-PLs). **(B)** Volume of each hepatic lobule in healthy or fibrotic livers. **(C)** Ortho slice view of hepatic sinusoids in the hepatic lobules. The red color represents the sinusoids in the lobular region. **(D-E)** 3D volume view of hepatic sinusoids in the hepatic lobules. Z-steps, 2 µm. The represented lobular sinusoids are colored, and other sinusoids are shown in white. **(F)** Density of the hepatic sinus in each healthy or fibrotic lobule. **(G)** Enlarged view of a hepatic sinusoid in a hepatic lobule. **(H)** Skeleton of a hepatic sinusoid in a hepatic lobule. The yellow color represents the skeleton, the red color represents the sinusoidal edges in the loop structure, and the blue points represent the sinusoidal nodes in the loop structure. **(I)** Ratio of all sinusoidal edges to all sinusoidal nodes in each lobule. **(J)** Number of sinusoidal edges per unit volume of a hepatic lobule. **(K-L)** Quantitative analysis of the number of loop edges per unit volume of a hepatic lobule (K) or in each hepatic lobule (L). All the data were collected from three mice in each group. n = 11 in the WT-CL group, n = 11 in the WT-PL group and n = 10 in the CCl_4_ group. Error bars denote the SDs. All the data were obtained from three independent repeated experiments and were analyzed by unpaired T test. The thickness of the projections in A-C was larger than 1.2 mm.

We then investigated the structure of the hepatic sinusoid because it is important for understanding and modeling the structure of the hepatic vascular system. Because the sinusoids are densely arranged in lobules, the liver lobe was acquired at a high resolution of 1.19 × 1.19 × 2 μm^3^/voxel to extract and reconstruct the hepatic sinusoid structure in healthy and fibrotic livers (Figure 3, Supplementary movies 5-7). Importantly, the imaging data presented in Figure 3A show that the hepatic lobules in the healthy mouse liver can be well described by either the classical lobular feature or the portal lobular feature, whereas the fibrotic liver can be mainly described by the portal lobular feature rather than the classical lobular feature. Thus, we mainly compared the sinusoidal structures in the healthy classical lobule, healthy portal lobule and fibrotic portal lobule.

Imaging data showed that the average volume of the classical lobule was 0.17 mm^3^ and the volume of the sinusoid in the classical lobule was 0.012 mm^3^, accounting for 7% of the volume of the intact lobule (Figure 3B-E). Furthermore, the average length of the hepatic sinusoids in healthy livers was 24.91 ± 1.50 μm, the average radius of the hepatic sinusoids was 2.92 ± 0.30 μm, and the average tortuosity of the hepatic sinusoids was 1.13 ± 0.02 (Figure S7A-C). Except for a decrease in the lobular volume, the other parameters of the sinusoidal structure showed no significant difference between classical lobule and portal lobule. To extract the sinusoidal microcirculation in liver fibrosis, a mouse liver fibrosis model was induced by administered CCl_4_ injections over a 6-week period. Interestingly, the imaging data also showed that liver fibrosis induced an increase in the sinusoidal density (from 7.4% to 8.8%) in fibrotic portal lobules (Figure 3F). Whole-mount imaging of intact lobules showed that the average volume of fibrotic lobules was similar to that of healthy portal lobules, but 20% lower than that of healthy classical lobules (Figure 3B). Additionally, liver fibrosis induced a 19% decrease in the sinusoidal length, a 4.8% decrease in the sinusoidal radius and a 7.2% decrease in the sinusoidal tortuosity in the portal lobule (Figure S7A-C). These data reveal that liver fibrosis not only induces an increase in the sinusoidal number in the lobule but also alters the morphology of the sinusoidal structure.

### Liver fibrosis induced an increase in sinusoidal branches and loops in the lobule

Having extracted the structural parameters of sinusoids in intact lobules, we then investigated the topological structure of the hepatic sinusoids in healthy and fibrotic livers (Figure 3G-H). Interestingly, the ratio of the total sinusoid edges to the total sinusoidal nodes was 1.369 ± 0.059 in the healthy classical lobule and 1.411 ± 0.015 in the healthy portal lobule (Figure 3I), which indicates that the topology of the sinusoid does not have a binary tree-like topology (the ratio was close to 1). In the fibrotic lobule, the ratio was increased to 1.48 ± 0.021 (Figure 3I), which indicated that liver fibrosis promotes hepatic sinusoid formulation of a more complex topological structure. The increased number of sinusoid edges and nodes in the unit volume of fibrotic lobule also supported the notion that liver fibrosis induces a more complex topology structure (Figure 3J, Figure S7D). Importantly, the degree of sinusoid nodes in normal or fibrotic lobules followed a lognormal distribution, indicating that the sinusoids in the lobules prefer a random network topology (Figure S7E). Liver fibrosis induced an increase in high-degree nodes (more than 3) from 0.1 to 0.14 (Figure S7F), suggesting that the sinusoidal connectivity is more complex in fibrosis.

Furthermore, we found that the hepatic sinusoidal system was rich in loop structures, and the dense arrangement of the sinusoid loops constituted a honeycomb network in the hepatic lobules (Figure 3G-H). Additionally, the low clustering coefficient (0.038 ± 0.006) and the low network centralization (< 0.001) demonstrated that the sinusoids in the lobules prefer a decentralized network rather than a star-like network (Supplementary Table 1). Unlike the mouse microvascular structures in the neocortex, which predominantly exhibit four edges in loops 39, the sinusoid loops contained more complex edge numbers (Figure 3H). Importantly, the fraction of the number of loop edges per unit volume of the portal lobule (Figure 3K) was significantly increased by 55% in the 4-edge loop, 66% in the 5-edge loop, 75% in the 6-edge loop and 84% in the 7-edge loop in the fibrotic liver. The fraction of the number of loop edges in each fibrotic lobule (Figure 3L) also showed a significant increase in the 4-7 edge loop. In addition, the angle between connected hepatic sinusoids was highly heterogeneous, and the average angle was approximately 110° (Figure S8). The smallest angle among connected hepatic sinusoids was close to 75° on average (Figure S8).

Thus, the hepatic sinusoids in the lobules preferred a random and decentralized honeycomb network. The dense distribution of the sinusoid loops in the lobules increased the surface contact between endothelial cells and hepatocytes, which might provide a critical microenvironment for substance exchange between the bloodstream and hepatocytes. Liver fibrosis induced sinusoidal rearrangement with an increased sinusoidal branch and loop structure. These topological changes in the sinusoid further modulate the sinusoidal microcirculation in hepatic lobules.

### Spatial distribution of DCs/macrophages in healthy and fibrotic lobules

**Figure 4:**
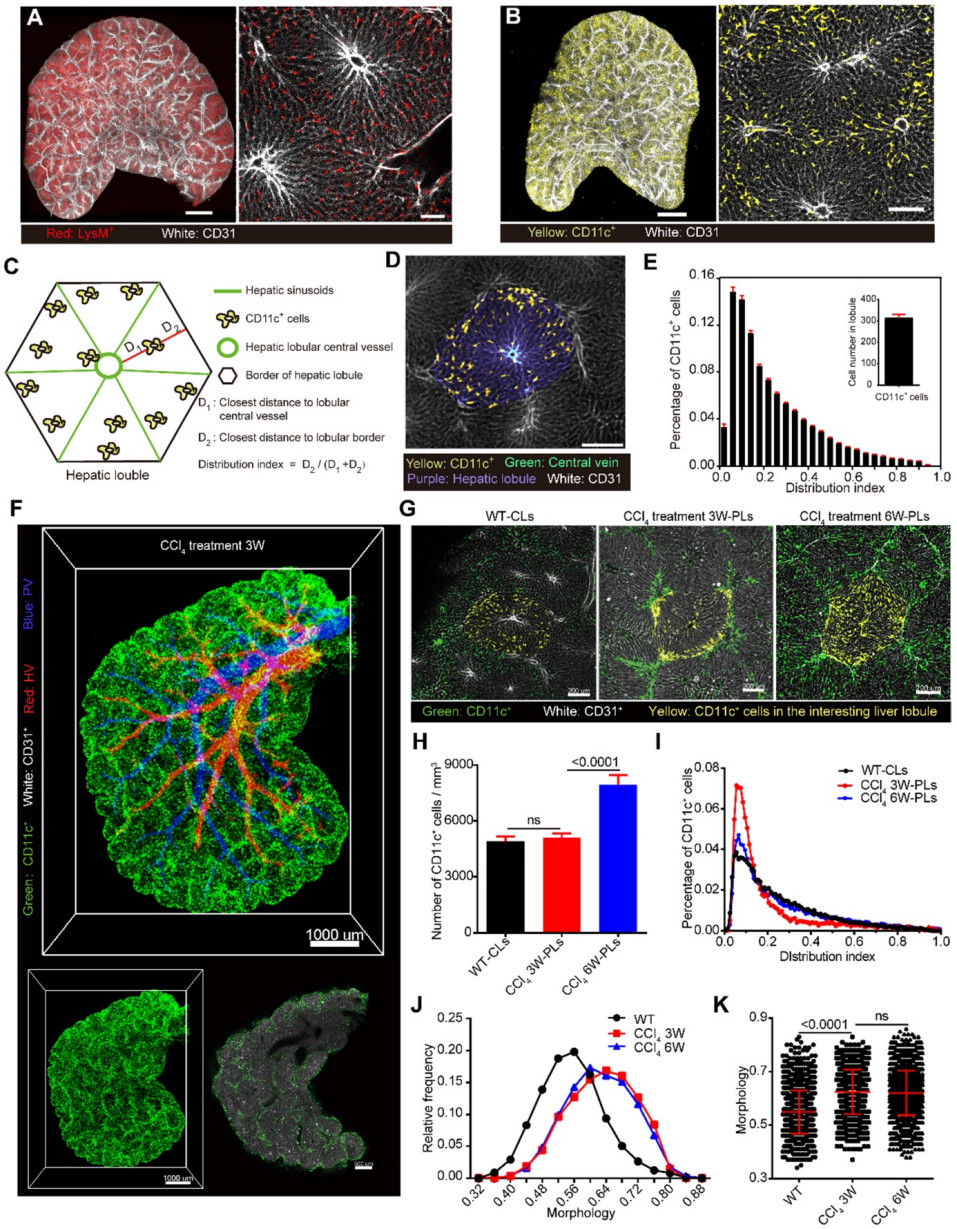
** CD11c^+^ cell aggregation at the lobular boundary in chronic liver injury and fibrosis. Liver vascular imaging after labeling with AF647-CD31Ab (white). (A-B)** Intact liver lobes of LysM-RFP and CD11c-YFP mice. LysM^+^ cells are shown in red, and CD11c^+^ cells are shown in yellow. Scale bar: 1000 µm. High-magnification views of the distribution of hepatic sinusoids and immune cells are shown on the right. Scale bar: 200 µm. Z-steps, 26.3 µm. **(C)** Schematic diagram of the distribution of CD11c^+^ cells in hepatic lobules. **(D)** Fluorescence imaging of the hepatic lobule monolayer segmented from (B). CD11c^+^ cells (yellow), CV (cyan), hepatic lobule (purple), and sinusoids (white). **(E)** Distribution of CD11c^+^ cells in each distribution index; the bin value is 0.04. The inset shows the calculated number of CD11c^+^ cells in a single hepatic lobule. Error bars denote the SEMs. **(F)** Whole-mount imaging of CD11c^+^ cells in the CCl_4_-induced fibrotic lobe. CD11c^+^ cells (green), HV (red), PV (blue), and sinusoids (white). Scale bar: 1000 µm. A single-slice view of the distribution of CD11c^+^ cells is shown on the right. Scale bar: 800 µm. Z-steps, 26.3 µm. **(G)** Fluorescence images of the monolayer hepatic lobule segmented from 3D data (1.2 × 1.2 × 1 mm^3^) of the WT group (left, WT-CLs), chronic liver injury (middle, CCl_4_ 3W-PLs), and liver fibrosis (right, CCl_4_ 6W-PLs). CD11c^+^ cells (green), CD11c^+^ cells in the interesting lobule (yellow), and sinusoids (white). **(H)** Normalization analysis of the number of CD11c^+^ cells in the hepatic lobule. The error bar denotes the SEM. **(I)** Distribution of CD11c^+^ cells in each distribution index; the bin value is 0.05. **(J)** Relative frequency of CD11c^+^ cells under different morphology indexes. **(K)** Quantitative analysis of the degree of sphericity of CD11c^+^ cells; each dot represents a cell. The error bar denotes the SD. **(E, H-K)** All the data were obtained from three independent repeated experiments and were analyzed by unpaired T test. The thicknesses of the projections in a, b, and f are 2.42 mm, 2.68 mm, and 2.50 mm, respectively.

The liver has a variety of immune cell components, which constitute the complex immune microenvironment of the liver 40, 41. The simultaneous visualization of immune cells and the vasculature would help us perform a comprehensive analysis of the liver structure and understand the spatial location of immune cells. The livers from LysM-RFP mice or CD11c-YFP mice were then cleared and imaged to acquire mesoscale information on the vasculature, macrophage and DC distribution in intact liver lobes (Figure 4A-B, Supplementary movie 4).

Having confirmed that 80% of CD11c-YFP cells in the liver are CD11c^+^ DCs through FACS (Figure S9), we then extracted the YFP^+^ cell distribution in CD11c-YFP mouse livers to study the spatial localization and distribution characteristics of DCs. Here, we defined the distribution index *ρ* as 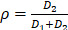 to characterize the DC distribution. *D_1_* represents the distance between CD11c^+^ cells and the lobular central vessel, and *D_2_* represents the distance between CD11c^+^ cells and the boundary of the hepatic lobule (Figure 4C-D). The distribution index ranges from 0 to 1, and a value close to 0 indicates that the cells are close to the boundary of the hepatic lobules. A quantitative analysis of the CD11c^+^ cells in 51 hepatic classic lobules revealed an average of 322 ± 19 CD11c^+^ cells in a single hepatic lobule in CD11c-Venus mice (Figure 4E). Among these CD11c^+^ cells in hepatic lobules, 2.2% were distributed surrounding the CV (*ρ* = 1), and 40% of CD11c^+^ cells were distributed near the hepatic lobular boundary (*ρ* < 0.15) (Figure 4E, Figure S10A-B, Supplementary movies 8). Interestingly, the distribution index of the CD11c^+^ cells from normal mice followed a lognormal distribution in the hepatic lobules that skewed toward the boundary and the presinusoidal space of the hepatic lobule (Area I) (Figure S9C-F). These results suggest that the boundary and presinusoidal space of the hepatic lobule might be a critical region for the capturing and processing of antigens by DCs in the liver.

Additionally, 3-week and 6-week periods of CCl_4_ injections were performed to extract CD11c^+^ cells in chronic liver injury and fibrosis. Imaging data confirmed that the number of CD11c^+^ cells per unit volume of a portal lobule in chronic liver injury showed no significant increase compared with that found for normal mice (classic lobule), and the distribution index of the CD11c^+^ cells also followed a lognormal distribution (Figure 4F-I). In contrast to normal mice, the percentage of CD11c^+^ cells located at the lobular boundary (*ρ* < 0.15) was 60% after 3-week CCl_4_ treatment and then decreased to 40% after 6-week CCl_4_ treatment (Figure 4I, Supplementary movie 9). In addition, the degree of DC sphericity increased from 0.54 to 0.63 after 3-week CCl_4_ treatment and to 0.62 after 6-week CCl_4_ treatment (Figure 4J-K), indicating that DCs reduce their dendrites and take on a spherical shape at the fibrotic lobular boundary in early fibrosis. Flow cytometric data and immunostaining data confirmed that DCs that aggregated at the inflammatory region showed upregulated CD86 and MHC-II expression (Figure S11). These results indicated that the aggregated DCs at the portal lobular boundary are mature DCs in CCl_4_-induced chronic injury. Recent studies showed that DCs form interstitial clusters in peripheral organs, promoting a regional immune response 42. The rearrangement of CD11c^+^ DCs at the portal lobular border before the formation of liver fibrosis suggests that DCs might enhance hepatic inflammation and fibrogenesis by activating hepatic stellate cells (HSCs) and T cells at the portal lobular border region.

### Spatial distribution of lobular DCs in NASH

Recently, DCs were reported to be as an important driver of non-alcoholic steatohepatitis (NASH) 43, 44. However, the distribution of DCs in intact fat lobule is unclear. Here, we performed a Methionine/Choline Deficient (MCD) diet -induced mouse model of NASH and investigated the DC distribution in fatty liver. The HE staining of liver section confirmed a typical feature of enlarged hepatic vacuolization in the MCD diet-induced fat liver, which suggesting that the NASH model was successfully performed (Figure S12). The transparent imaging data of the fat liver show that the number of CD11c^+^ cells per unit volume of hepatic lobules increased by 125% after 3 weeks of MCD diet feeding (Figure S12A-C). Furthermore, we calculated the distribution distance of CD11c^+^ cells from the CV. The average distance from CD11c^+^ cells to central vein in healthy lobule was 221.7 ± 103.7 µm (means ± SD) and this distance decreased to 127.3 ± 69.3 µm (means ± SD) in fat lobule. Fifty-two percent of CD11c^+^ cells in fat lobule distributed within the 120 µm range from the CV, while the value was 18% in healthy lobule. Thus, CD11c^+^ cells in the NASH model were preferentially distributed around the CV when compared with the healthy liver (Figure S12D). The immunofluorescent imaging of the bile duct in fat liver sections by CK19 labeling also showed that CD11c^+^ cells did not accumulate at the portal regions (Figure S12F). Thus, during the development of NASH, a large number of CD11c^+^ cells infiltrate the liver and distributed around the CV.

### CX3CR1^+^CCR2^+^ macrophages surround liver micrometastases in a mouse model of colorectal liver metastasis

**Figure 5:**
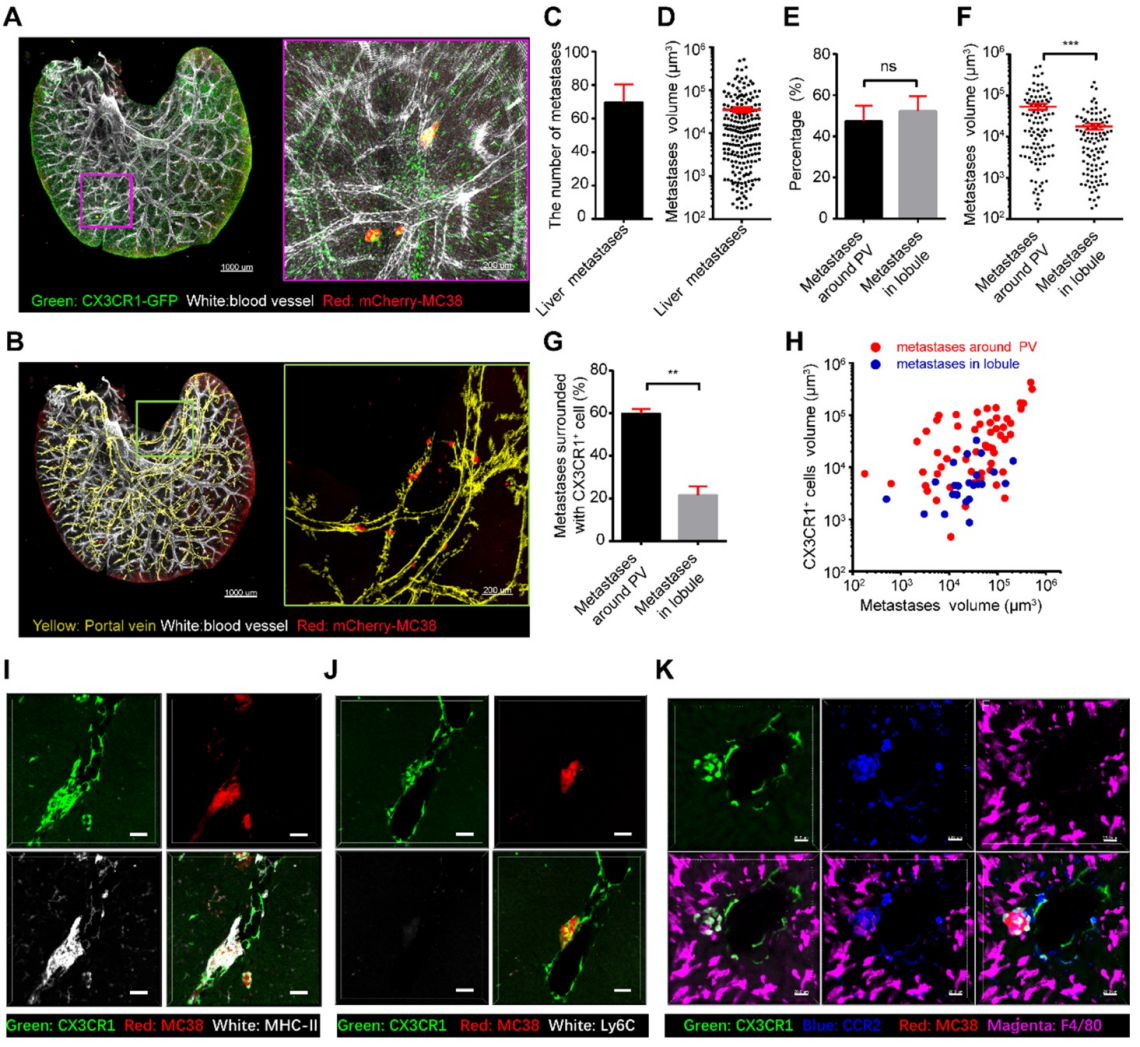
** CX3CR1^+^ cells surround liver metastases in the portal area. (A)** 3D confocal imaging of the liver lobe with colorectal micrometastases of CX3CR1-EGFP mice. Scale bar, 1000 µm. The data in the red box are enlarged. Scale bar, 200 µm. Z-steps, 26.3 µm. **(B)** Imaging of micrometastases in the liver caudate lobe. White, vasculature of CX3CR1-EGFP mice labeled with anti-mouse CD31; Red, mCherry-MC38; Yellow, liver PV. Scale bar, 1000 µm. The data in the red box are enlarged. Scale bar, 200 µm. The thickness of the projections in a-b is 2.40 mm. **(C)** Average number of micrometastases in the liver caudate lobe. **(D)** Mean volume of micrometastases in the liver caudate lobe. **(E-F)** Percentage and mean volume of micrometastases around the liver PV and in the hepatic lobule. **(G)** Percentage of micrometastases surrounded by CX3CR1^+^ cells around the liver PV and in the hepatic lobule. **(H)** Relationship between the micrometastasis volume and the volume of CX3CR1^+^ cells surrounding metastases located around the liver PV (red) or in the hepatic lobule (blue). Each point represents one metastasis. **(I-K)** Immunofluorescence imaging of liver sections stained with anti-MHC-II, anti-Ly6C, anti-CCR2 and anti-F4/80 antibodies (white). CX3CR1^+^ cells are shown in green. Scale bar: 50 µm. **(C-I)** All the data were obtained from three independent repeated experiments; ***P < 0.001, ****P < 0.0001, Mann-Whitney U test.

The liver is an organ that is highly prone to colorectal metastasis 45, 46. Here, we established a visible liver metastasis model to study the crosstalk between CX3CR1^+^ macrophages and colorectal metastatic cells through the splenic injection of mCherry-MC38 cells into CX3CR1-EGFP mice (Figure S13). Confocal imaging of the MC38-bearing liver lobes showed that CX3CR1^+^ cells surrounded some micrometastases near the PV in the liver on the third day after tumor cell injection (Figure 5A-D, Supplementary movie 10), suggesting a close relationship between CX3CR1^+^ cells and micrometastasis. Quantitative data showed that half of the liver metastases were located around the PV, and the others were located in the hepatic lobules (Figure 5E). Interestingly, the volume of micrometastases near the PV was (5.39 ± 0.88) × 10^4^ μm^3^, which is three times larger than that in hepatic lobules (Figure 5F). Importantly, 59.86% of the liver metastases at the PV were surrounded by CX3CR1^+^ cells; however, only 21.56% of the liver metastases in the hepatic lobules contained CX3CR1^+^ cells (Figure 5G). In addition, the volume of micrometastases with surrounding CX3CR1^+^ cells was 6.74 ± 1 × 10^4^ μm^3^, which was 4.75 times larger than the volume of micrometastases without surrounding CX3CR1^+^ cells (Figure S13A). The volume of CX3CR1^+^ cells around each tumor metastasis distributed in the PV was 14 times larger than that in the hepatic lobule (Figure 5H, Figure S13B). Furthermore, immunofluorescence staining data showed that the CX3CR1^+^ cells that surrounded the micrometastases around the portal region were MHC-II^+^, F4/80^+^, CCR2^+^, and Ly6C^-^ (Figure 5I-K). Interestingly, the proportion of M2 macrophages and the M2/M1 ratio was slightly increased at day 3 in early colorectal liver metastases (Figure S14). In conclusion, these results reveal that the micrometastatic microenvironment of colorectal cells is heterogeneous in different regions of the liver, and CX3CR1^+^CCR2^+^F4/80^+^ macrophages around colorectal micrometastases might contribute to the colonization and expansion of metastatic cells at the early stage of tumor metastasis.

### Colorectal cell-secreted CCL2 is critical for the recruitment of CX3CR1^+^CCR2^+^F4/80^+^ macrophages to the liver

**Figure 6:**
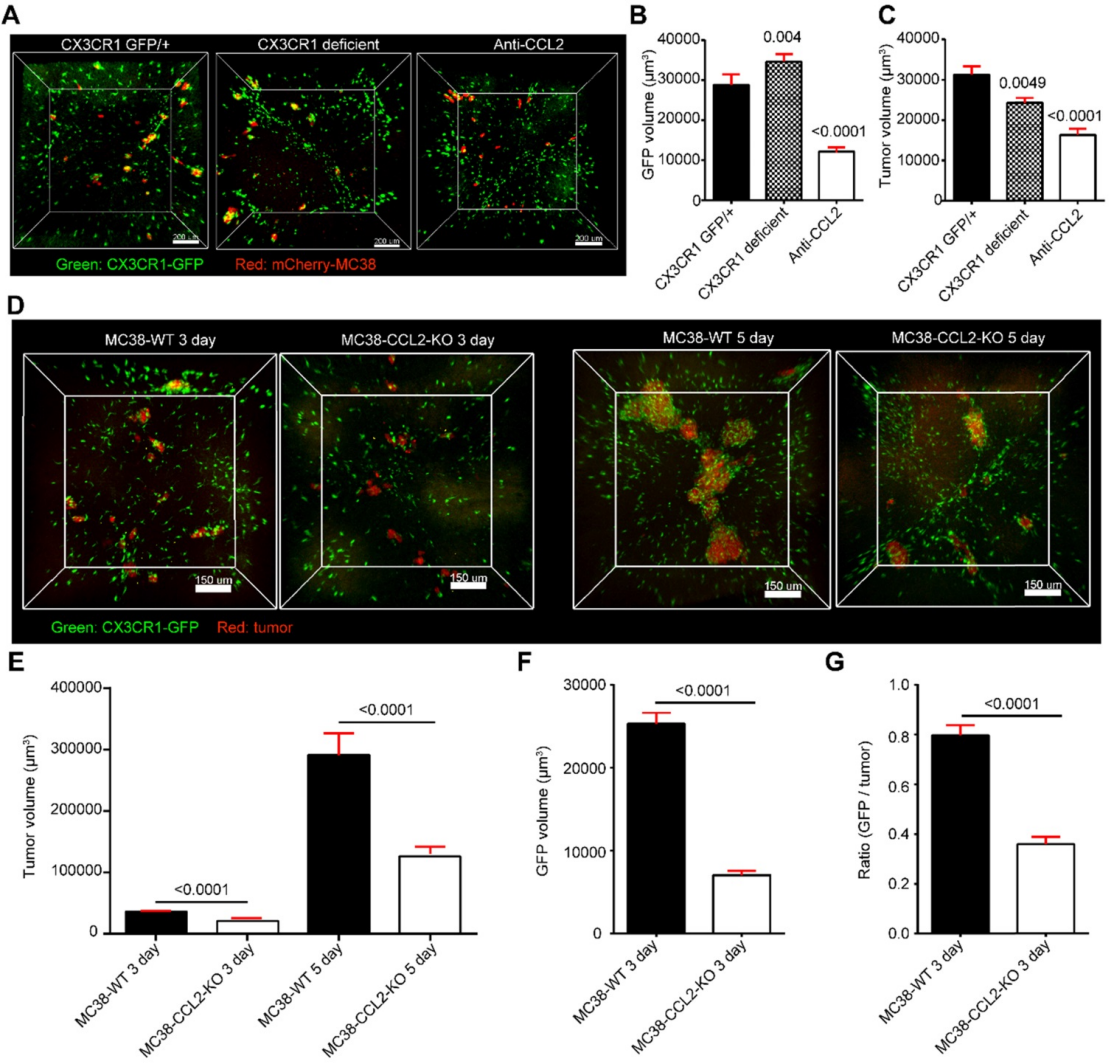
** Identification of the immunomodulation factors for recruiting CX3CR1^+^CCR2^+^ macrophages to liver micrometastases. (A)** 3D confocal imaging of liver micrometastases in CX3CR1^gfp/+^ heterozygous mice, CX3CR1-deficient mice and anti-CCL2 blockade mice. **(B-C)** The mean volume of CX3CR1^+^ cells around liver micrometastases and mean volume of liver micrometastases in CX3CR1^gfp/+^ heterozygous mice (n = 207), CX3CR1-deficient mice (n = 351) and mice with anti-CCL2 blockade (n = 129). The imaging data were collected from at least 9 image regions of three mice in E-G. **(D)** 3D confocal imaging of the liver at 3 and 5 days after intrasplenic inoculation of MC38-WT or MC38-CCL2-KO cells in CX3CR1^gfp/+^ mice. Scale bar: 150 µm. **(E)** Mean volume of metastases at 3 and 5 days after intrasplenic inoculation of MC38-WT or MC38-CCL2-KO cells. **(F)** Mean volume of CX3CR1^+^ cells around micrometastases and **(G)** ratio of CX3CR1^+^ GFP around the unit micrometastases at 3 days after intrasplenic inoculation of MC38-WT or MC38-CCL2-KO cells. n = 572 in the MC38-WT group and n = 756 in the MC38-CCL2-KO group at 3 days after intrasplenic inoculation. n = 280 in the MC38-WT group and n = 234 in the MC38-CCL2-KO group at 5 days after intrasplenic inoculation. The error bar denotes the SEM. Mann-Whitney U test. The n of each group indicates the number of analyzed micrometastases. A tumor with a diameter less than 0.2 mm was defined as a micrometastasis. The thickness of the projections in each region is larger than 0.5 mm. The imaging data were collected from at least 26 image regions of four mice in E-G.

We then sought to uncover the key factor for eliciting the recruitment of CX3CR1^+^CCR2^+^F4/80^+^ macrophages. Imaging data showed that the volume of recruited CX3CR1^gfp/+^ cells around colorectal micrometastases was decreased by 75% after anti-CCL2 antibody treatment (Figure 6A-B). However, the volume of recruited CX3CR1^gfp/gfp^ cells in CX3CR1-deficient mice did not differ from that of CX3CR1^gfp/+^ cells in CX3CR1 heterozygous mice (Figure 6A-B). Thus, the CCL2/CCR2 axis, not the CX3CL1/CX3CR1 axis, promotes CX3CR1^+^CCR2^+^ macrophage recruitment during early liver micrometastasis. Furthermore, the volume of colorectal micrometastases displayed a 60% decrease after CCL2 blockade compared with that obtained with IgG treatment, suggesting that CCL2 contributes to colorectal cell colonization in the liver (Figure 6C).

To investigate whether colorectal cells direct mobilized CX3CR1^+^CCR2^+^ F4/80^+^ macrophage recruitment through CCL2, we generated CCL2-knockout MC38 cell lines (MC38-CCL2-KO) using CRISPR-Cas9 gene editing (Figure 6D, Figure S15). The volume of micrometastases in the MC38-CCL2-KO group was reduced by 42.4% at day 3 and by 56.7% at day 5 compared with that in the MC38-WT group (Figure 6E). Here, we defined a recruitment coefficient σ as σ = (GFP volume) / (tumor volume) to quantify the ability of colorectal cells to recruit CX3CR1^+^CCR2^+^ macrophages per unit tumor volume. The quantitative data shown in figure 6F confirmed that the volume of CX3CR1^+^CCR2^+^ macrophages around micrometastases at day 3 was 72.2% lower than that found for the MC38-CCL2-KO group. In addition, the recruitment coefficient of the MC38-CCL2-KO group was 45%, which was lower than that of the MC38-WT group (Figure 6G). Together, these results reveal that colorectal cells recruit CX3CR1^+^CCR2^+^F4/80^+^ macrophages at liver micrometastases by secreting CCL2. These results suggest that metastatic colorectal cells might shape a favorable micrometastatic environment through CCL2-mediated CX3CR1^+^CCR2^+^F4/80^+^ macrophage recruitment.

## Discussion

Acquiring an intact map of the liver vasculature and immunocytes is a potent supplement to the data obtained by flow cytometry and tissue sectioning when studying the structure and function of the liver. Here, using our developed liver-CUBIC method for the whole-mount imaging of intact liver lobes, we successfully acquired the fine vasculature structure and the spatial distribution of DCs/macrophages in an intact liver lobe at single-cell resolution (Graphical Abstract).

A variety of transparency techniques, including SeeDB 47, uDISCO 48, CUBIC 30, Clarity 47, and PEGASOS 49, can effectively achieve transparency of intact organs and even intact mice. Nevertheless, compared with other organs, such as the brain and thymus, transparency of the liver is achieved more slowly and is less satisfactory because the liver is rich in fat and connective tissue. A recent study showed that the double resin casting micro-computed tomography (DUCT) approach combined with micro-computed tomography (µCT) achieves good imaging of the bile duct in an intact liver lobe 50. However, limited by the resin viscosity, the resolution of DUCT can only reach 5 µm/pixel which is not enough for large volume imaging of bile canaliculi and immune cells in the liver. In our method, based on the defatting step in liver clearing, liver-CUBIC improved the retention of the fluorescent signal in the liver and achieved clearer and deeper images, which greatly promoted acquisition of the 3D structure from intact liver lobe data. The advantages of the liver-CUBIC method, including the compatibility of fluorescent protein-based endogenous labeling and immunofluorescence labeling and the shorter transparency time, make this method more suitable for 3D imaging of intact liver lobes (approximately 10 × 10 × 2.3 mm^3^). Limited by the working distance of the objective lens for confocal imaging, the imaging depth of the liver sample is controlled to within 3 mm when using a 10× objective lens. Thus, all our imaging data were collected from caudate lobes. Higher-resolution data of other liver lobes can be obtained in the future through advanced optical microscopy approaches, such as fluorescent micro-optical sectioning tomography technology 51.

Recently, imaging data based on liver perfusion and micro-CT scanning have revealed the anatomical structure and topological characteristics of the hepatic macrocirculation. However, the structure of the sinusoidal microcirculation in the hepatic lobules remains unclear. Some studies have cleared small-sized liver tissues (0.332 × 0.332 × 0.170 mm^3^) and explored the topology information of the hepatic sinusoids through CUBIC 23, whereas spatial structure information of the hepatic sinusoids in the intact hepatic lobule is still missing. Based on the liver-CUBIC method, we characterized the distribution features of the hepatic lobules in an intact liver lobe and defined the arrangement of the sinusoids in separated lobules. The spatial information acquired from cleared liver lobes confirmed that the hepatic sinusoids exhibited a random and decentralized honeycomb network structure in hepatic lobules and that adjacent hepatic sinuses tended to form complex loop structures. Liver fibrosis not only induced an increase in the sinusoidal density but also promoted significant changes in the topological structure of hepatic sinusoids. Among them, the probability of sinusoidal branch points with high connectivity and the probability of forming a loop structure significantly increase. These changes may further influence the flow direction of blood in lobules, reduce the fluidity of blood in hepatic sinusoids, and increase the vascular pressure of the interlobular vein and PV. The observed increase in the sinusoidal density and the detected change in the topological structure may be important factors for inducing portal hypertension in clinical liver fibrosis. The published literature indicates that sinusoidal distortion in liver fibrosis is critical for promoting the progression of fibrosis, and this distortion is mainly caused by sinusoidal angiogenesis 52-54. Our whole-mount imaging of sinusoids in intact lobules revealed that fibrosis not only increased the sinusoidal number but also altered the sinusoidal topology. A recently published paper also supports the notion that sinusoid capillarization in liver fibrosis is vascular remodeling rather than angiogenesis 4. Therefore, our study of the hepatic sinusoid topology will improve our understanding of the hemodynamics of the hepatic circulatory system and the metabolic microenvironment of hepatic lobules in healthy and fibrotic livers.

A cell localization and subpopulation analysis based on the hepatic vascular structure is one of the most attractive applications of liver-CUBIC. Benefitting from the segmentation of the hepatic lobules in the intact liver, we determined the 3D distribution of CD11c^+^ DCs/macrophages in healthy and fibrotic lobules. Previous confocal imaging studies have found that DCs surround the hepatic sinusoid 16 but could not characterize the distribution of DCs in the whole hepatic lobule. Our data confirmed that CD11c^+^ DCs prefer to distribute in the presinusoidal space of the hepatic classical lobules, which is close to PTs 11. These results suggest that the presinusoidal space of the hepatic lobule might provide a pivotal microenvironment for antigen capture by DCs in the lobule when the antigens flow into the hepatic lobules. During the process of early liver fibrosis, the liver-CUBIC approach uncovered the rearrangement of hepatic CD11c^+^ cells at the portal lobular boundary with a spherical shape after chronic liver injury. In the steady state, hepatic DCs mainly perform tolerogenic responses to antigens. During liver fibrosis, DCs exhibit immunogenic responses to antigens and govern hepatic inflammation by stimulating NK cell, T cell, and HSC activation 55. The arrangement of CD11c^+^ DCs at the lobular border at the early stage of liver fibrosis suggests that DCs might enhance hepatic inflammation and fibrogenesis at the border region. The molecular and cellular mechanisms that mobilize DC redistribution in the lobule region need further investigation.

The liver is an organ that is highly prone to tumor metastasis. Through whole-mount imaging of liver lobes, we found that CX3CR1^+^CCR2^+^ macrophages are associated with the formation and expansion of micrometastases. Previously, Lei Zhao *et al*. reported that metastatic colorectal cell-secreted CCL2 promotes CD11b/Gr1^mid^ myeloid cell recruitment at day 7 in macroscopic liver metastases 56. Jiao Zheng *et al*. found that the CX3CL1/CX3CR1 axis promotes macrophage accumulation and survival in liver metastases 57. However, the key immunomodulatory factor for recruiting immune cells and promoting the colonization of colorectal cells at the early stage of liver metastases is unclear due to the lack of whole-mount imaging data of liver lobes. Here, we confirmed that metastatic colorectal cells promoted CX3CR1^+^CCR2^+^ macrophage recruitment to the micrometastatic region through CCL2. The migration pathway of CX3CR1^+^CCR2^+^ macrophage to the colorectal micrometastatic region might contain two pathways: one might be the circulating monocyte transendothelial migrating through the hepatic sinus 58, and the other might be liver CCR2^+^ macrophage migrating from the parenchymal regions 59. The function of recruited CX3CR1^+^CCR2^+^ macrophages might be mainly to maintain the homeostasis of liver immune tolerance in the early liver metastasis model.

Compared with CT, MRI, PET, and other non-invasive imaging technology, the optical transparency imaging approach recently has not allowed clinical diagnosis without surgery. At present, this method is mainly suitable for basic biological research on model animals. In the future, we believe that the optical transparency imaging approach can be further applied to 3D imaging of liver biopsy, especially in the identification of the tumor boundaries to improve the surgical rationality and the evaluation of the tumor invasiveness in the clinical diagnosis of early-stage hepatocellular carcinoma.

## Conclusions

In summary, mesoscale visualization of the intricate cross-scale vasculature and the DC/macrophage distribution in intact liver lobes at single-cell resolution through whole-mount imaging of the liver lobe based on liver-CUBIC provides a novel platform for understanding the hemodynamics of the hepatic circulatory system, the metabolic microenvironment and the immune surveillance network of the liver. The application of this advanced strategy for the analysis of the anatomical structure of liver diseases and for addressing immune-pathogen crosstalk in the liver will profoundly broaden the avenues for the future study of liver physiology and pathology.

## Supplementary Material

Supplementary methods, figures, tables, movie legends.

Supplementary movie 1.

Supplementary movie 2.

Supplementary movie 3.

Supplementary movie 4.

Supplementary movie 5.

Supplementary movie 6.

Supplementary movie 7.

Supplementary movie 8.

Supplementary movie 9.

Supplementary movie 10.

## Abbreviations

HA: hepatic artery
PV: portal vein
HV: hepatic vein
CV: central vein
PTs: portal triads
3D: three-dimensional
CT: computed tomography
MRI: magnetic resonance imaging
PFA: paraformaldehyde
AF647: Alexa Fluor 647
CCl_4_: carbon tetrachloride
