# Supplementary material for: Mesoscale visualization of three-dimensional microvascular architecture and immunocyte distribution in intact mouse liver lobes
Source: Theranostics. 2022 Jul 11;12(12):5418–33. doi: 10.7150/thno.71718 (PMC9330531; doi:10.7150/thno.71718)
Supplement: Supplementary file 1 — Supplementary methods, figures, tables, movie legends. [file thnov12p5418s1.pdf]

Supplementary Materials for

Title: Mesoscale visualization of three-dimensional microvascular architecture and immunocyte distribution in intact mouse liver lobes

Zheng Liu[1,3], Mengli Xu [1,3], Songlin Huang[1,3], Qi Pan[1], Chong Liu[1], Fanxin Zeng[1], Zhan Fan[1], Yafang Lu[1], Jialu Wang[1], Jinxin Liu[1], Xinlin Li[1], Qingming Luo[1,2,] *, Zhihong Zhang[1,2,] *

[1] Britton Chance Center and MoE Key Laboratory for Biomedical Photonics, Wuhan National Laboratory for Optoelectronics-Huazhong University of Science and Technology, Wuhan, Hubei 430074, China
[2] School of Biomedical Engineering, Hainan University, Haikou, Hainan 570228, China
[3] These authors contributed equally: Zheng Liu, Mengli Xu, Songlin Huang
* Corresponding authors: Zhihong Zhang, Qingming Luo.
Email: czyzzh@mail.hust.edu.cn (Zhihong Zhang), qluo@hainanu.edu.cn (Qingming Luo)

Supporting Method

Reagents

CUBIC-L was used for tissue delipidation and contained 10% (w/v) N-butyldiethanolamine (Macklin, CAS: 102-79-4) and 10% (w/v) Triton X-100 (Sigma, CAS: 9036-19-5) in water. CUBIC-R was used for tissue RI matching and contained 45% (w/v) antipyrine (Macklin, CAS: 60-80-0) and 30% (w/v) nicotinamide (Macklin CAS: 98-92-0) in water. Lipase and PFA were purchased from Sigma.

Mouse model of NASH

The methionine-choline deficient (MCD) diet was used for a mouse model of nonalcoholic steatohepatitis (NASH). The adult mice were fed the MCD diet (10 g of diet per 20 g body weight), and the diet was changed every two days. The mouse livers were harvested after 3 weeks of feeding.

Generation of the MC38-CCL2-KO cell line

PX458-U6-empty was a gift from Prof. Feng Zhang (Addgene plasmid # 48138). sgCCL2-1 (tgctgttcacagttgccggc) and sgCCL2-2 (cgggtcaacttcacattcaa) were separately cloned into the PX458-U6-empty plasmid and the MIGR1-mAmetrine-U6-empty plasmid. MC38 cells were transfected with PX458-U6-sgCCL2-1 and MIGR1-mAmetrine-U6-sgCCL2-2 by Lipo2000 (Invitrogen, USA). Forty-eight hours later, EGFP- and mAmetrine-positive MC38 cells were sorted into 96-well plates as single cell per well. Two weeks later, the mouse CCL2 ELISA kit (purchased from 4A Biotech Co., Ltd., China) was used to identify the CCL2-KO cell line clone.

Flow cytometric analysis of CCL2 expression in MC38 cells

MC38 cells was cultured in 6-well plates to a density of 80%. Brefeldin A (BioLegend,

USA) was added to the culture medium, and the cells were cultured for 5 h at 37 ℃. The MC38 cells were then harvested in 0.25% trypsin/EDTA, washed twice with PBS and then subjected to fixation and permeabilization with a Fix/Perm kit (BioLegend, USA). The MC38 cells were then stained with APC-conjugated anti-mouse CCL2 (BioLegend, clone: 2H5) in the dark for 12 h at 4 ℃. After staining, MC38 cells were washed twice with Perm/Wash Buffer before flow cytometric analysis.

Immunofluorescence staining of frozen liver sections

To detect the state of $CD11c^+$ cells and the bile duct in CCl_4-induced fibrosis, the liver lobes were fixed with 4% PFA for 12-24 h at 4 °C and then dehydrated in 30% sucrose solution overnight. The dehydrated lobes were frozen in OCT (Sakura, Torrance, CA, USA) and cut into 20-μm sections using a freezing microtome (Leica, Germany). OCT was removed by three washes in PBS (8 min per wash), and the liver sections were then blocked with PBS/1% BSA/0.2% Triton X-100 for 1 h at room temperature. After blocking, the liver sections were separately stained with PE anti-mouse CD86 (BioLegend, clone: PO3), BV421 anti-mouse MHC-II (BioLegend, clone: M5/114.15.2), and rabbit anti-mouse CK19 (Abcam, clone: EP1580Y) overnight at 4 ℃. For CK19 staining, the liver sections were washed three times in PBS (8 min per wash) and then stained with Alexa Flour 647 goat anti-rabbit IgG (Invitrogen, clone: AB_2535813) as the secondary antibody for 3 h at room temperature. All liver sections were then washed three times in PBS (8 min per wash) and imaged with an LSM 710 confocal microscope (Zeiss, Germany).

The procedures of CUBIC and liver-CUBIC clearing protocol

The protocol for CUBIC is based on the previously published work [1] and the protocol for liver-CUBIC is described below.

Step-1 The anesthetized mice were first transcardially perfused with PBS (pH 7.4) for 10 min and then with 4% (w/v) PFA (pH 7.4) in PBS for 10 min at room temperature.

Step-2 The mice were transcardially perfused with approximately 100 mL of lipase saturation solution for 1 h at room temperature. For preparation of the lipase saturation solution, 200 mg lipase (Sigma, cat: L3126) was fully stirred for dissolving in 100 mL PBS within 1 h, and the solution was then centrifuged at 4000 rpm, 10 min, at room temperature. The supernatant was then filtered through with a 70-μm filter before perfusion.

Step-3 The mice were transcardially perfused with 4% PFA for 10 min at room temperature.

Step-4 The harvested organs (e.g., liver, spleen, kidney, and thymus) of mice were immersed in 4% PFA for more than 12 h, at 4 °C.

Step-5 The fixed organs were then washed three times (at least 2 h per wash) with PBS to remove PFA before clearing at room temperature.

Step-6 The livers were immersed in 50% CUBIC-L (mixed with water) for delipidation for more than 6 h under 50-60 rad/s shaking at 37 °C.

Step-7 The livers were immersed in CUBIC-L for 2 days (until the solution became clear) with shaking at 37 °C, and the solution was changed every 24 h.

Step-8 The livers were washed three times (more than 2 h every time) in PBS for volume retraction at room temperature.

Step-9 The livers were immersed in 50% CUBIC-R (mixed with water) for RI matching for more than 6 h at 4 °C.

Step-10 The liver lobes were immersed in CUBIC-R (the pH of CUBIC-R was adjusted to 9.5 with NaOH) for 1 day at 4 °C.

3D immunofluorescent staining protocol of liver-CUBIC

Step-1 The liver after delipidation with the CUBIC-L solution was collected and washed three times (more than 2 h each time) in PBS at room temperature.

Step-2 The liver was immersed in HEPES-TSC buffer (containing 10 mM HEPES, 200 mM NaCl, 0.5% casein, and 10% Triton X-100) with shaking for 1.5 h. At the same time, the antibody solution (containing primary antibodies and secondary antibodies) was diluted in HEPES-TSC with shaking for 1.5 h at room

temperature.

Step 3 The liver was immersed in the antibody dilution for 2 days at room temperature and 1 day with shaking at 4 °C for staining.

Step-4 The liver was washed twice in 0.1 M PB with 0.1% TritonX-100 for 30 min at room temperature.

Step-5 The liver was washed twice in 0.1 M PB for 1 h at room temperature.

Step-6 The liver was postfixed with 1% (w/v) PFA (pH 7.4) in PB overnight at room temperature.

Step-7 The liver was washed twice in 0.1 M PB (more than 1 h each time) at room temperature.

Step-8 The liver was immersed in 50% and 100% CUBIC-R for RI matching at room temperature.

3D immunofluorescent staining protocol of iDISCO

Step-1 The fixed liver was washed three times (more than 30 min each time) in PBS at room temperature.

Step-2 The liver was dehydrated through gradient methanol (20%, 40%, 60%, 80%, 100% and 100% diluted in H_2O); 1 h each concentration at room temperature.

Step-3 The liver was incubated in 33% methanol/66% DCM with shaking overnight at room temperature.

Step-4 The liver was washed twice in 100% methanol (more than 30 min each time) with shaking at room temperature.

Step-5 The liver was immersed in 5% H_2O_2 in methanol for 12 h at 4 °C.

Step-6 The liver was rehydrated with gradient methanol (80%, 60%, 40%, 20% diluted in H_2O; 1h at each concentration) and washed in PBS for 1 h at room temperature.

Step-7 The liver was washed twice in PBS+0.2% TritonX-100 (1 h each time) at room temperature.

Step-8 The liver was incubated in permeabilization solution (0.3 M Glycine+20%

DMSO+ 0.2% TritonX-100 in PBS) for 1 day, at 37 °C.

Step-9 The liver was blocked in blocking solution (6% fetal bovine serum+10% DMSO+0.2% TritonX-100 in PBS) for 1 day at 37 °C.

Step-10 The liver was incubated in the primary antibody (rabbit anti-mouse TH 1:500) diluted in PBS with 3% fetal bovine serum+5% DMSO+0.2% Tween-20 for 2 days at 37 °C.

Step-11 The liver was washed 5 times (more than 2 h each time) in PBS+0.2% Tween-20.

Step-12 The liver was incubated in the second antibody (goat anti-rabbit AF647 1:500) diluted in PBS with 3% fetal bovine serum+0.2% Tween-20 for 2 days at 37 °C.

Step-13 The liver was washed 5 times (more than 2 h each time) in PBS+0.2% Tween-20.

Step-14 The liver was dehydrated with gradient methanol (20%, 40%, 60%, 80%, 100%, and 100% diluted in H_2O; 1h each concentration) at room temperature.

Step-15 The liver was incubated in 33% methanol/66% DCM with shaking for 3 h at room temperature.

Step-16 The liver was immersed in DiBenzyl Ether (sigma, 108014) for RI matching.

Vessel labeling

We labeled the mouse blood vessels with Alexa Fluor 647 (AF647)-CD31Ab (BioLegend, Clone: MEC13.3). The CD31 antibody (15 μg) was diluted in PBS to a total volume of 200 μL and then injected *via* the tail vein. Subsequently, Alexa Flour 647-CD31Ab (5 μg) was injected into the tail artery of mice to distinguish the HA. Ten min later, the mice were anesthetized with a mixture of 2% (w/v) chloral hydrate and 10% (w/v) urethane (90 μL/10 g) by intraperitoneal (i.p.) injection.

Confocal imaging

For imaging of the cleared liver lobe, an LSM 780 NLO confocal microscope (Zeiss,

Germany) equipped with a 10× water immersion objective (N.A. 0.45) and 32 anode Hybrid-GaAsP detector was used. First, the 50% CUBIC-R solution containing 2% agarose (defined as CUBIC-agarose solution) was heated in a microwave oven until the liquid boiled and the agarose was dissolved completely. Next, 5 mL of CUBIC-agarose solution was poured into a petri dish (55 mm in diameter and 17 mm in depth). When the gel temperature decreased to approximately 37 ℃, the RI-matched liver lobe was placed into the CUBIC-agarose solution. The CUBIC-agarose solution did not need to flood the sample. When the CUBIC-agarose solution solidified, the transparent sample was embedded in a petri dish and then covered with 3-5 mL of CUBIC-R solution for imaging with an LSM780 microscope equipped with a 10× objective.

For imaging and segmentation of the HV, PV, and HA, hepatic lobules, DCs/macrophages, and liver micrometastasis, 3D imaging of an intact liver lobe was performed at 26.3-µm z-steps in a field of approximately 10 × 10 × 2.3 mm^3, and the imaging conditions were adjusted according to the fluorescence intensity of different samples. The microscope zoom was 1×. For segmentation and reconstruction of the hepatic sinusoid structure, imaging was performed at 2-µm z-steps in a field larger than 1.2 × 1.2 × 1.2 mm^3. The microscope zoom was set to 0.7×. Spectral imaging and linear unmixing were used to distinguish autofluorescence, vascular tissue and immune cells.

To compare the retention of the fluorescence signal (YFP and RFP) after liver clearing between liver-CUBIC and CUBIC, imaging was performed at 5-µm z-steps in a field of 1024 × 1024 pixels, and the imaging conditions were kept consistent for different samples without spectral imaging and linear unmixing. The microscope zoom was set to 1×. The mean fluorescence intensity of YFP and RFP cells in the liver lobes was further investigated and calculated using Imaris software. For imaging of immunostained liver sections, an LSM 710 confocal microscope (Zeiss, Germany) equipped with a 20× dry immersion objective (N.A. 0.8) was used. Images were acquired over a z-range deeper than 20 µm in 3-µm z-steps. The excitation wavelengths were 405 nm for DAPI and BV421, 488 nm for GFP and YFP, 561 nm for tdTomato,

PE and Alexa Fluor 594 (AF594), and 633 nm for AF647 and APC.

Vessel and hepatic lobule segmentation

All segmentation of hepatic vessels and lobules was performed using Imaris software (Bitplane, Belfast, UK). In brief, the AF647-CD31Ab-labeled vessels were first automatically segmented and reconstituted using the "Surfaces" module with "absolute intensity" thresholding. Based on the existing vascular architecture of the HV, PV, HA, and CV obtained by micro-CT, the liver blood vessels are composed of three components, namely, the HV, the PV, and the HA. In the branched structure of large blood vessels, the HV has the largest average diameter, followed by the PV, and the diameter of the HA is significantly smaller than that of the liver venous system. In addition, the liver CV is located in the center of the liver lobular structure and is the branch of the HV. Thus, according to these features, the HV, PV, and HA structures were further manually segmented using "Mask Properties" in "Surface". The hepatic lobule was manually segmented according to the distribution of the liver PV and HV (the PV was distributed at the edge of the hepatic lobule, and the branch of the HV was located in the center of the hepatic lobule) using the "Surfaces" module in Imaris.

Quantification of the $CD11c^+$ cell spatial distribution in the hepatic lobules

We analyzed the distribution of $CD11c^+$ cells in 97 hepatic lobules in the caudate lobe of CD11c-Venus mice. First, we analyzed the distance between $CD11c^+$ cells and the CV of the hepatic lobule using Imaris software; the cells less than 20 μm from the CV were defined as $CD11c^+$ cells distributed around the hepatic CV. Quantification of the proportions of these cells was then performed using GraphPad Prism. In addition, we calculated the spatial distribution index of the remaining $CD11c^+$ cells in the hepatic lobules. As shown in Figure 5, the distribution index was calculated as $\rho = \frac{D_2}{D_1+D_2}$, where D_1 is the distance between $CD11c^+$ cells and the central vessel of hepatic lobules and D_2 is the distance between $CD11c^+$ cells and the boundary of hepatic lobules.

According to the calculated distribution index, we calculated the logarithm of the distribution index and the probability of $CD11c^+$ cells and simulated the distribution of $CD11c^+$ cells through exponential growth, Gaussian and lognormal models using GraphPad Prism. The distribution of $CD11c^+$ cells under different bin values (bin = 0.02, 0.04, 0.06, 0.08, and 0.1) was verified.

Topological analysis of the hepatic sinusoids

To quantitatively analyze the topology of the hepatic lobules, the cleared lobes were imaged in a volume (X-Y-Z) larger than 1.2× 1.2 × 1.2 mm^3 at a resolution of 1.19 × 1.19 × 2 μm^3/voxel size (X-Y-Z). The imaging data were first processed with a "Gaussian filter" using Imaris, and the lobule regions were then manually segmented based on the location of the CV, sinusoids and PTs. The sinusoids were then automatically segmented and processed through binarization using Imaris with "background subtraction" thresholding. The binarized sinusoid data were then processed with "AutoSkeleton" in Amira software (Thermo Fisher) to extract the sinusoid network parameters in the lobules. For additional network analysis, the exported parameters were processed and analyzed using Network Analyzer (Cytoscape 3.7.2).

Flow cytometry

The mice were first perfused with PBS to remove blood from the liver. The livers were then collected, cut into pieces, and subjected to enzymatic digestion at 37 ℃ with 0.5 mg/mL collagenase type IV (Worthington, LS004188) and 0.1 mg/mL Liberase^{TM} TL (Roche, 5401020001) in DMEM for 60 min. The enzymatically digested liver tissue was then triturated in DMEM and filtered through 70-μm cell strainers. After two washes with PBS, the liver cell pellets were resuspended in PBS, added to 10 mL of 36.5% Percoll and centrifuged for 20 min at 1000×g. The liver cell pellets at the bottom of the Percoll gradient were collected and washed twice with PBS for 5 min at 500×g. To detect the percentages of DCs and macrophages, the cells were labeled with the

Fixable Viability Dye eFluor™ 780 (Invitrogen, Cat: 65-0865) and the antibodies PerCPCy5.5 anti-mouse MHC II (BioLegend, clone: M5/114.15.2), BV605 anti-mouse CD64 (BioLegend, clone: X54-5/7.1), and BV421 anti-mouse CD11c (BD Pharmingen, clone: M418) at room temperature for 15 min. To detect the state of DCs in CCl_4-induced fibrosis, the cells were labeled with the antibodies BV510 anti-mouse MHC-II (BioLegend, clone: M5/114.15.2), AF647 anti-mouse CD80 (BioLegend, clone: 16-10A1), APC anti-mouse CD86 (BD Pharmingen, clone: GL-1), and APC anti-mouse CD40 (BioLegend, clone: 3/23) at room temperature for 15 min. PI was added to the cell suspension before analysis. To detect CD206 and CD80 expression in CX3CR1-GFP macrophages, the cells were labeled with eFluor 780 and the antibodies BV421 anti-mouse F4/80 (BD Pharmingen, clone: T45-2342), PE anti-mouse CD80 (BioLegend, clone: 16-10A1), and APC anti-mouse CD206 (BioLegend, clone: C068C2) at room temperature for 15 min. The details of the antibodies are shown in Supplementary Table 3. The liver cells were then subjected to two 5-min washes with 3 mL of PBS. The liver cell suspension was analyzed with a CytoFLEX flow cytometer (Beckman Coulter, USA). The data were analyzed using FlowJo software (FlowJo, Ashland, OR, USA).

Statistical analysis

GraphPad Prism was used for the statistical analyses. The error bars denote the SDs or SEMs. Unpaired t test or Mann–Whitney U test was used for comparisons of groups. Significant differences between the groups are indicated as follows: ns for no significant difference, * for $P < 0.05$; ** for $P < 0.01$; *** for $P < 0.001$; and **** for $P < 0.0001$.

Supplementary figures and tables

Figure S1 Quantitative analysis of fluorescence preservation under different liver depths and transparency methods.

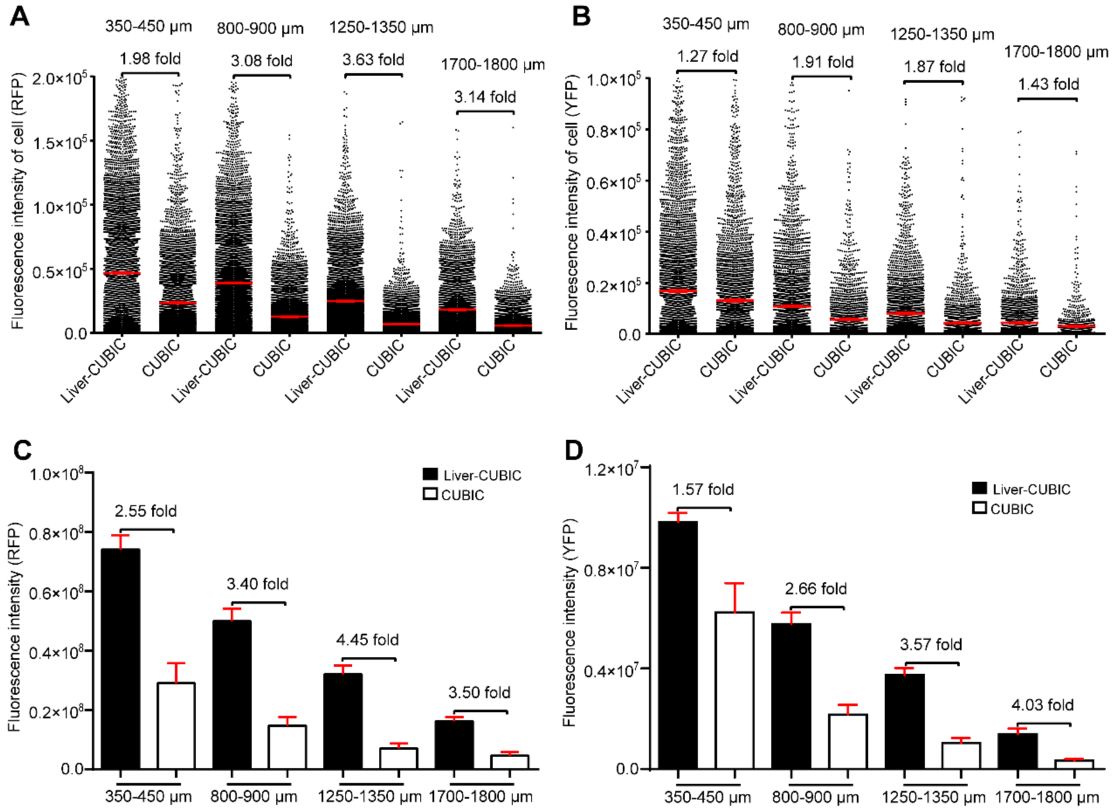

Figure S1. Quantitative analysis of fluorescence preservation under different liver depths and transparency methods. (A-B) Fluorescence intensity of each cell at different liver depths and with different transparency methods. Each dot represents the fluorescence intensity of one RFP^+ or YFP^+ cell at each depth. These data show that the average RFP intensity of each cell in the liver-CUBIC group was 1.98-3.14-fold higher than that in the CUBIC group, and the average YFP intensity of each cell in the liver-CUBIC group was 1.27-1.91-fold higher than that in the CUBIC group. The data were collected from 9 image regions. (C-D) The total RFP and YFP fluorescence intensity of cells in the liver-CUBIC group was 2.55- to 4.45-fold and 1.57- to 4.03-fold higher than that in the CUBIC group, respectively (n=9 measurements per group). The error bars denote the SEMs; Mann–Whitney U test.

Figure S2 Investigation of the influence of pH and temperature conditions on GFP fluorescence retention.

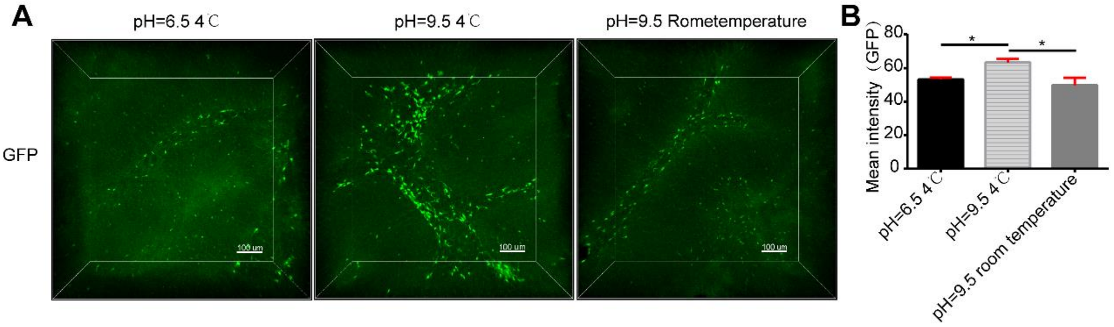

Figure S2. Investigation of the influence of pH and temperature conditions on GFP fluorescence retention. (A) Confocal imaging of 0.5-mm-thick liver slices from 9-week-old CX3CR1-EGFP mice under different pH and temperature conditions. Scale bar: 100 μm. (B) A liver slice under the “pH=9.5 4 ℃” condition showed the best fluorescence preservation; unpaired T test.

Figure S3 3D imaging of mouse organs after liver-CUBIC clearing.

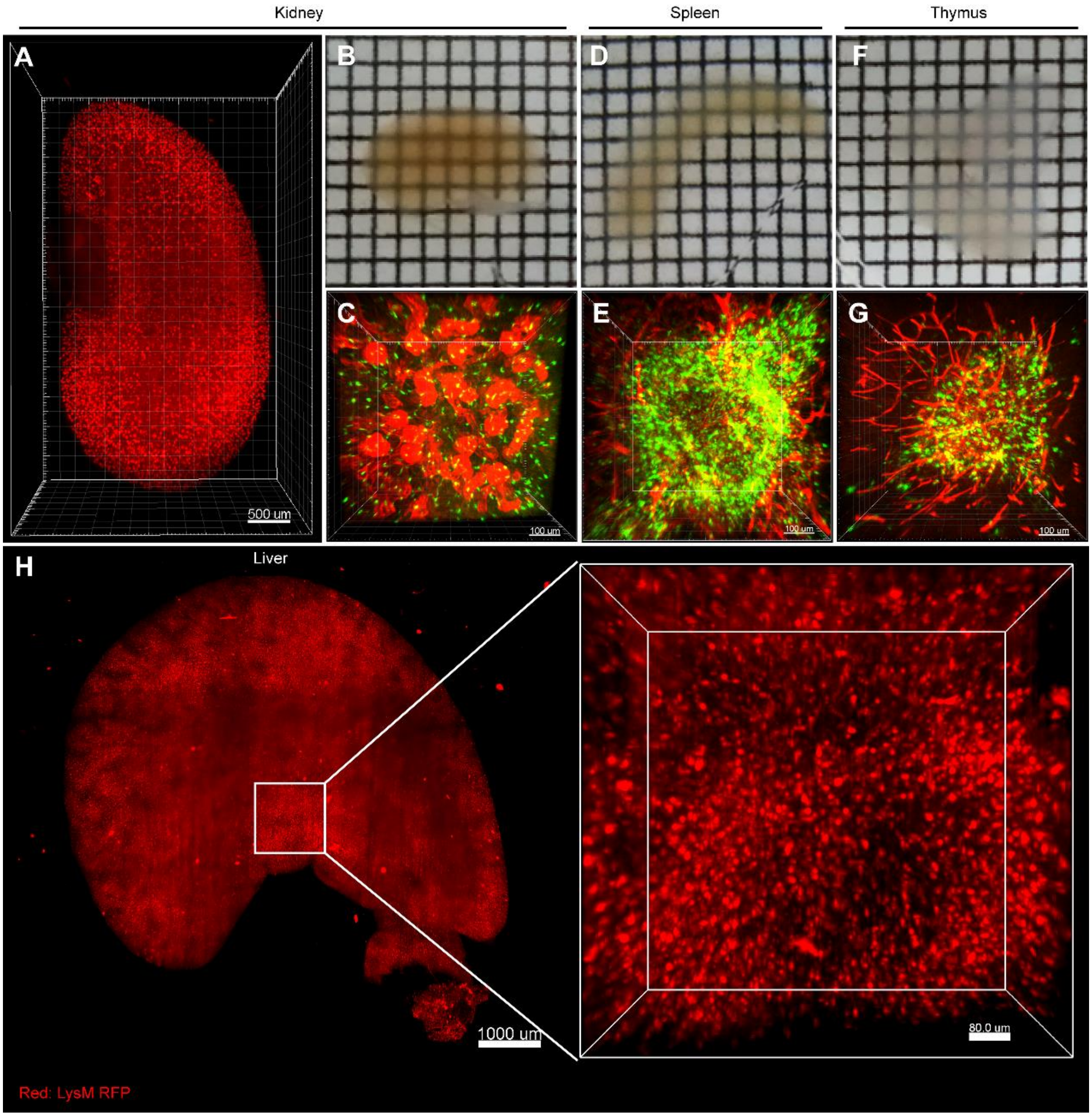

Figure S3. 3D imaging of mouse organs after liver-CUBIC clearing. (A) 3D reconstruction of the kidney glomeruli of 4-week-old CX3CR1-EGFP female mice by light-sheet fluorescence microscopy (LSM, Ultramicroscope, LaVision BioTec, Germany). Anti-mouse AF647-CD31Ab (red) was used to label the kidney. The LSM was equipped with an sCMOS camera (Andor Neo) and a 2×/0.5 objective lens with a dipping cap. The excitation wavelength for AF647 was 633 nm. For entire scanning of the organ, the cleared kidney was immersed in CUBIC-R in the sample reservoir, and the voxel size was 2 × 2 × 5 um^3. Scale bar: 500 μm. (B, D, F) Bright-field images of the kidney, spleen, and thymus from CX3CR1-EGFP ×mTmG mice (aged 8-9 weeks, female); the size of the grid is 1.6 mm × 1.6 mm. (C, E, G) Confocal imaging of (B, D, F). Green, CX3CR1 GFP^+ cells; Red, vascular tissue with the tdTomato reporter protein. Scale bar: 100 μm. (H) 3D imaging of LysM-RFP^+ cells in intact liver lobe with LSM. The LysM-RFP^+ cells are shown in red. Scale bar: 1000 μm. High-magnification views of the distribution of immune cells are shown on the right. Scale bar: 80 μm. The LSM system, which was developed by the Fei group, was used for liver lobe imaging of LysM-RFP^+ cells. The liver lobe (8.7× 8.7 × 3 mm) was imaged by an MVX10 microscope equipped with a 4× objective. In addition, a dual-side Bessel light sheet with a thickness of 5 μm was used. The scanning voxel resolution was 1.625 μm×1.625 μm×3 μm, and the scanning excitation light was 561 nm.

Figure S4 3D immunostaining of the liver lobe by liver-CUBIC clearing.

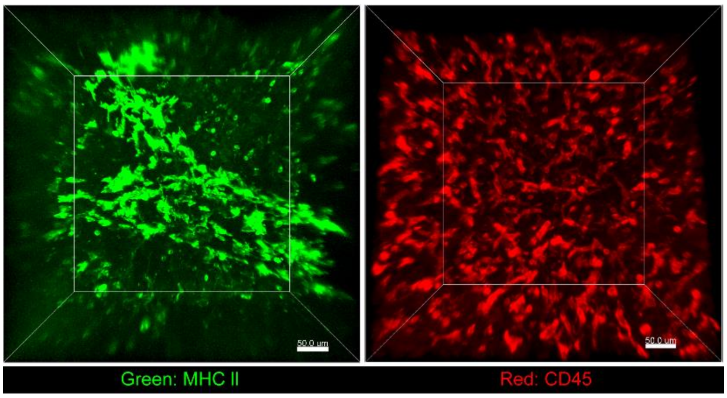

Figure S4. 3D immunostaining of the liver lobe by liver-CUBIC clearing. Staining of MHC-II-positive cells (left) and CD45-positive cells (right) in the liver.

Figure S5 Comparison of liver-CUBIC and iDISCO in 3D immunostaining.

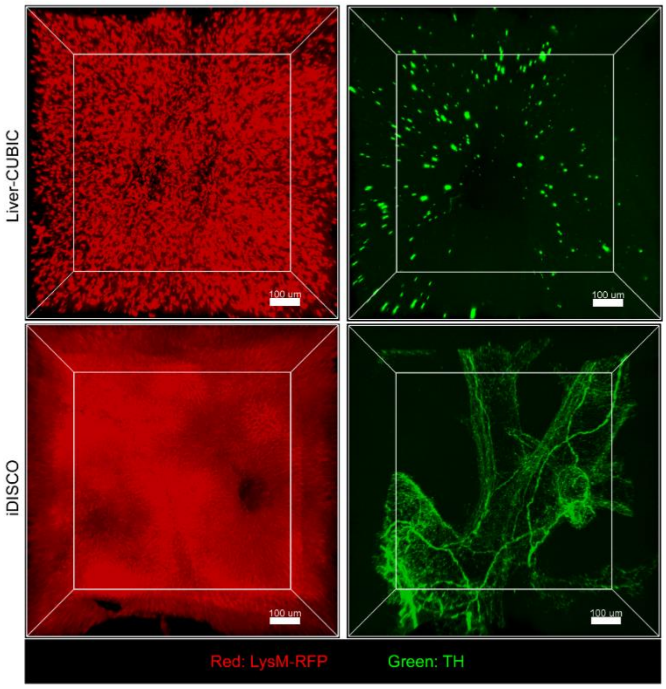

Figure S5. Comparison of liver-CUBIC and iDISCO in 3D immunostaining. 3D immunostaining of the liver lobe by liver-CUBIC (up) and iDISCO (down) clearing approach. TH nerves are shown in green. LysM RFP$^+$ cells are shown in red. Scale bar: 100 µm.

Figure S6 Vascular imaging and segmentation of the HA in intact liver lobes.

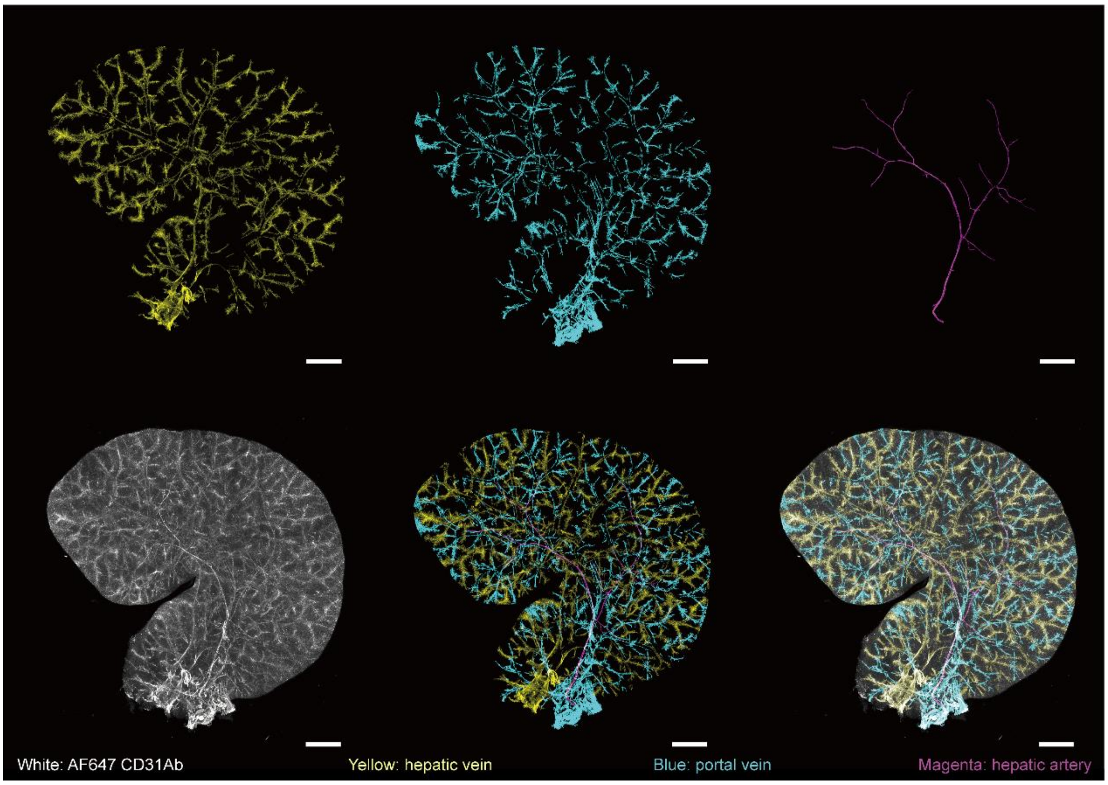

Figure S6. Vascular imaging and segmentation of the HA in intact liver lobes. The liver vasculature was labeled with AF647-CD31Ab (white). HV (yellow), PV (blue) and HA (magenta). Scale bar: 1000 µm. Z-steps, 26.3 µm.

Figure S7. Topology of the hepatic sinusoid system.

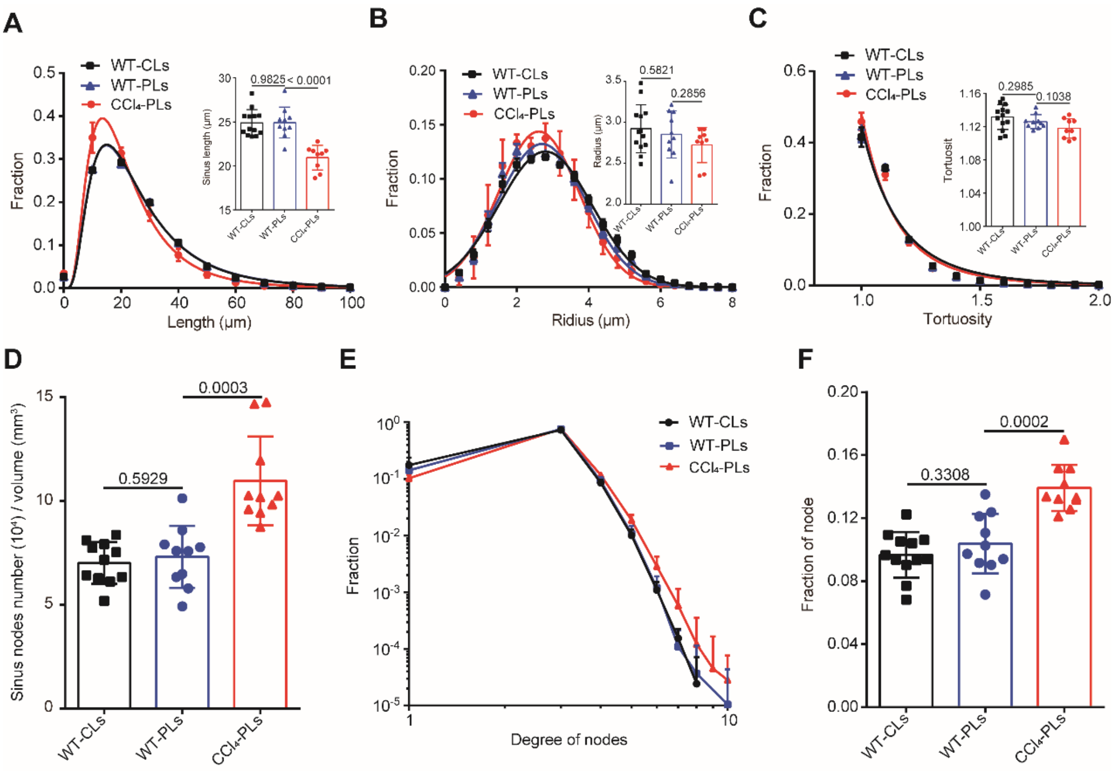

Figure S7. Topology of the hepatic sinusoid system. (A-C) Quantitative analysis of the length (A), radius (B) and tortuosity (C) of hepatic sinusoids in healthy or fibrotic lobules. (D) Number of sinusoidal nodes per unit volume of a hepatic lobule. (E) Probability distribution of the sinusoidal node degree in hepatic lobules. (F) Sum fraction of a sinusoidal node degree higher than 3 in E. All the data were obtained from three independent repeated experiments; unpaired T test.

Figure S8 Smallest angle among hepatic sinusoids in healthy lobules

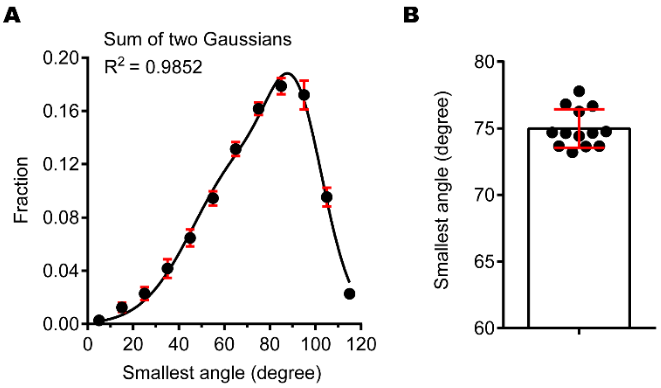

Figure S8. Smallest angle among connected hepatic sinusoids in healthy lobules. The branching angles for connected sinusoids with a coordination number of three were collected to analyze the smallest angle. (A) The probability distribution of the smallest angle followed a sum of two Gaussians. (B) Average value of the smallest angle from 13 hepatic lobules. The error bars denote the SDs.

Figure S9 Percentages of DCs and macrophages in CD11c-YFP cells

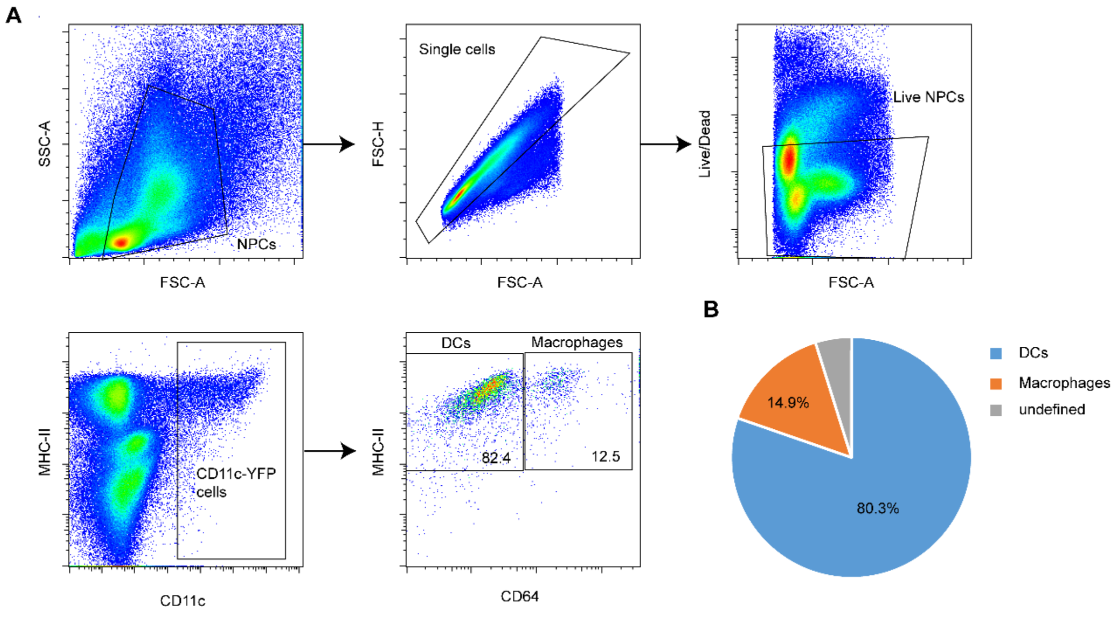

Figure S9. Percentages of DCs and macrophages in CD11c-YFP cells. (A) Gating strategy for DCs and macrophages in liver nonparenchymal cells (NPCs). (B) Average percentages of DCs and macrophages in CD11c-YFP cells. n = 3 mice. $CD11c^{+}$ DCs were gated as $MHC\text{-}II^{+}CD64^{-}$, and $CD11c^{+}$ macrophage lines were gated as $MHC\text{-}II^{+}CD64^{-}$.

Figure S10 Spatial distribution of CD11c^{+} cells in hepatic lobules

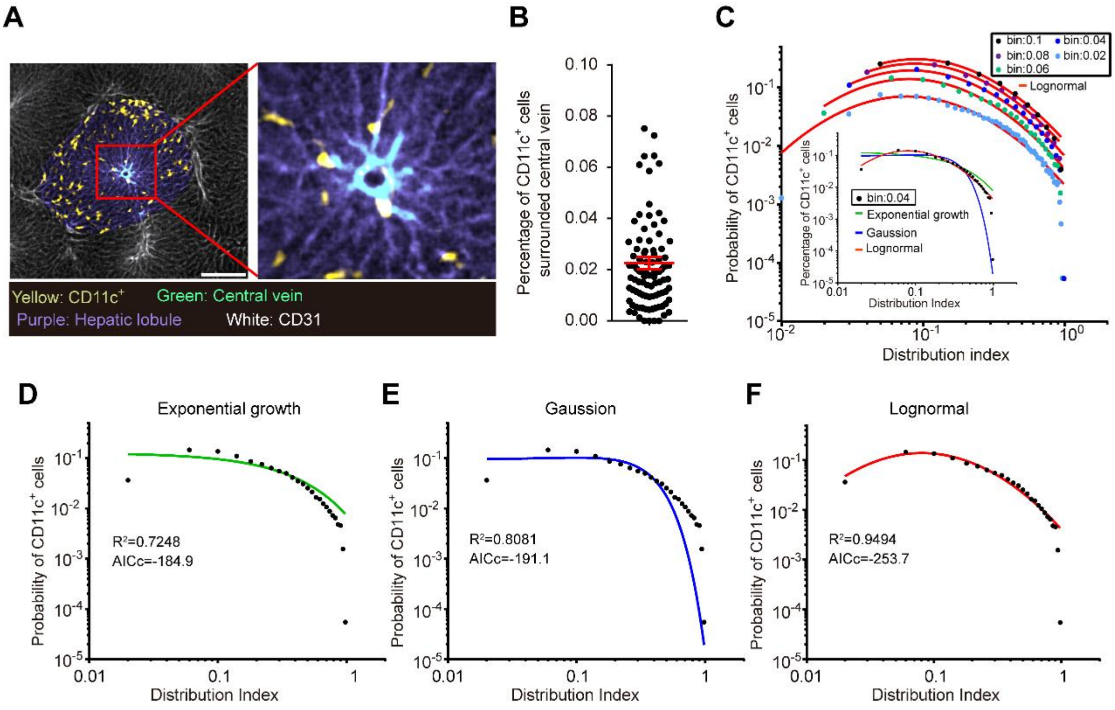

Figure S10. Spatial distribution of CD11c^{+} cells in hepatic lobules. (A) Representative fluorescence imaging of CD11c^{+} cells in a hepatic lobule monolayer. CD11c^{+} cells (yellow), CV (cyan), hepatic lobule (purple), and sinusoids (white). (B) Quantitative analysis of the percentage of CD11c^{+} cells surrounding the CV. Each dot represents a hepatic lobule. The error bar denotes the SEMs. (C) Lognormal probability distribution of CD11c^{+} cells under different bin values. The illustration shows the comparison of the lognormal model with exponential growth and Gaussian model fitting curves when the bin value was 0.04. (D-F) Comparison of lognormal with exponential growth and Gaussian model fitting curves when the bin value was 0.04. The trend lines and the values of R^2 and Akaike information criterion (AIC) showed that CD11c^{+} cells in the hepatic lobules conformed to a lognormal distribution: $y = a \times e^{-0.5(\frac{\ln(\frac{\rho}{b})}{c})^2}$. In the function, a represents the height of the center of the distribution in y units, b represents the value of ρ at the peak of the distribution, and c represents the measure of the width of the distribution.

Figure S11 Detection of the state of DCs in CCl_4-induced chronic injury

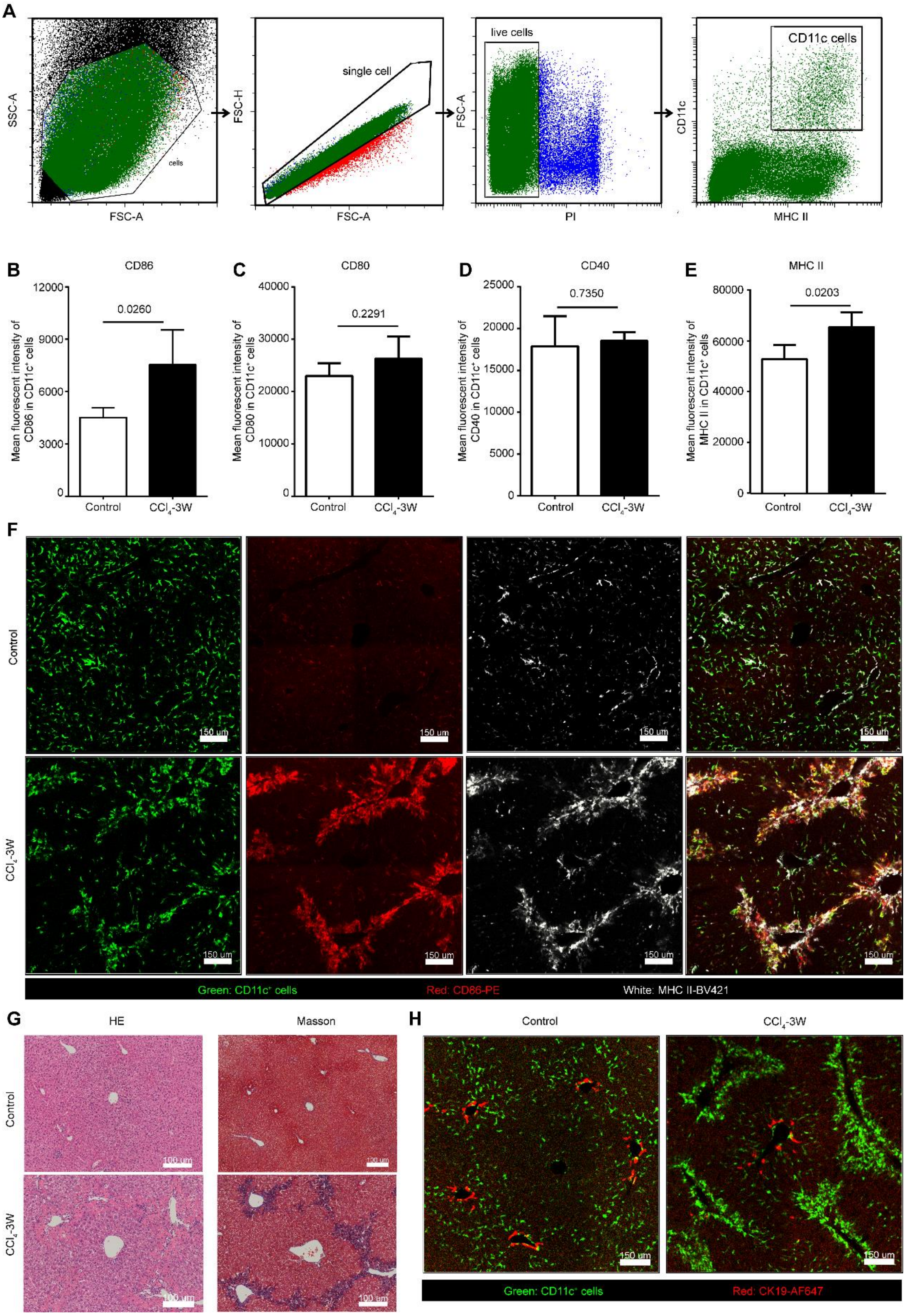

Figure S11. Detection of the state of DCs in CCl_4-induced chronic injury. (A) Gating strategy for DCs in liver NPCs. (B-E) The mean fluorescent intensity of CD86, CD80, CD40 and MHC-II in $CD11c^+$ cells, n = 4 mice. The error bars denote the SDs. (F) Immunofluorescence imaging of liver sections stained with PE anti-mouse CD86 (red) and BV421 anti-mouse MHC-II (white). $CD11c^+$ cells are shown in green. Scale bar: 150 μm. (G-H) HE and Masson staining of liver sections of a normal liver (up) and a CCl_4-injured liver (down). Scale bar: 100 μm. (H) Immunofluorescence imaging of liver sections stained with anti-CK19 (red). CD11c+ cells are shown in green. Scale bar: 150 μm.

Figure S12 Spatial distribution of DCs in hepatic lobules in the NASH model

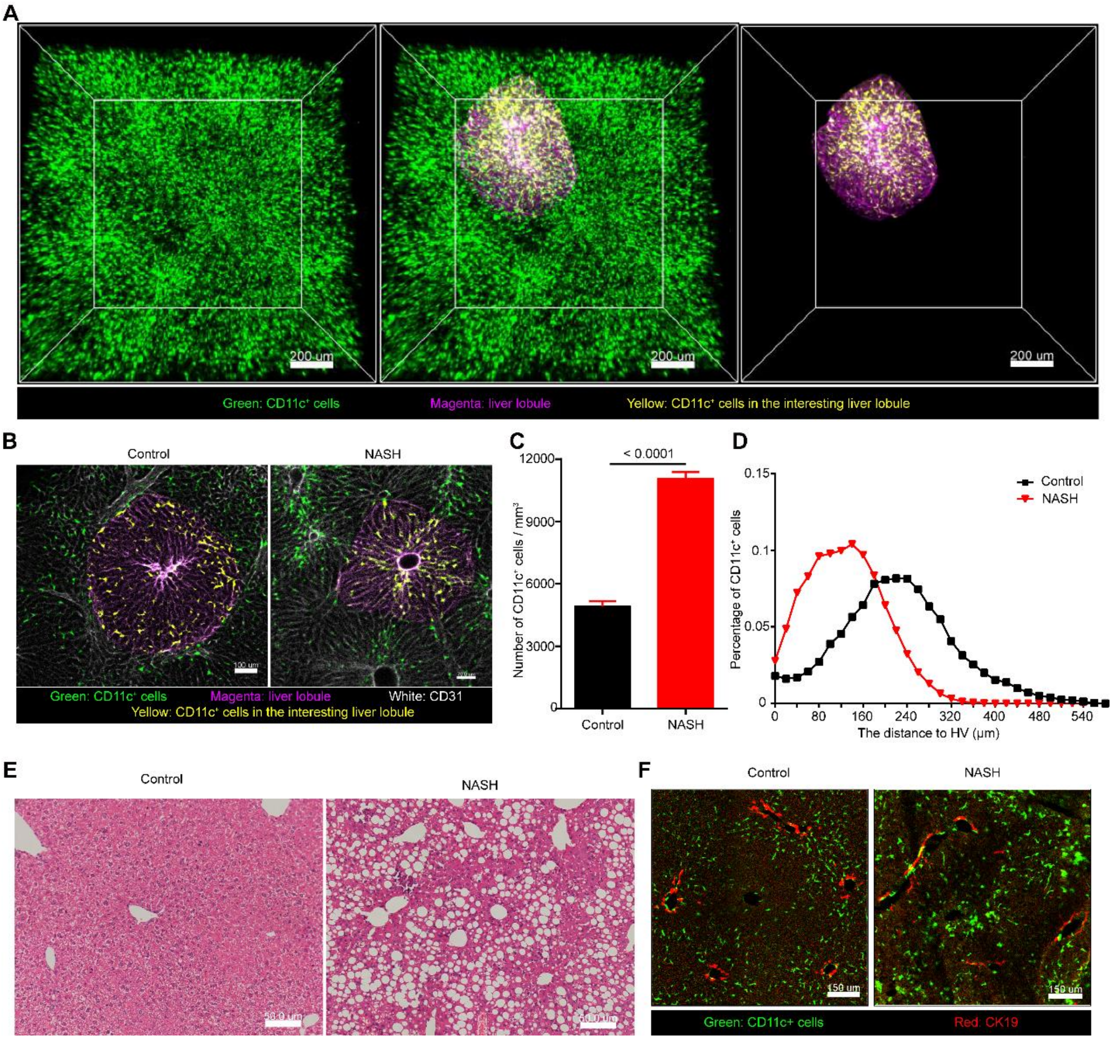

Figure S12. Spatial distribution of DCs in hepatic lobules in the NASH model. (A) 3D confocal imaging of the NASH liver of CD11c-venus mice. Scale bar, 200 μm. Green: CD11c$^+$ cells, magenta: a representative hepatic lobule in the imaging region, yellow: CD11c$^+$ cells in hepatic lobules. (B) Fluorescence images of the monolayer hepatic lobule from 3D imaging data (1.2 × 1.2 × 1 mm^3) of a normal liver (left) and a NASH liver (right). CD11c$^+$ cells (green), CD11c$^+$ cells (yellow) in the interesting lobule (magenta), and sinusoids (white). Scale bar: 100 μm. (C) Normalization analysis of the number of CD11c$^+$ cells in hepatic lobules. The error bar denotes the SEM. (D) Distance of CD11c$^+$ cells to the CV in hepatic lobules. (E) HE staining of liver sections of a normal liver (left) and a NASH liver (right). Scale bar: 50 μm. (F) Immunofluorescence imaging of liver sections stained with anti-CK19 (red). CD11c$^+$ cells are shown in green. Scale bar: 150 μm.

Figure S13 Quantitative analysis of the mean volume of tumor metastases and CX3CR1$^+$ cell

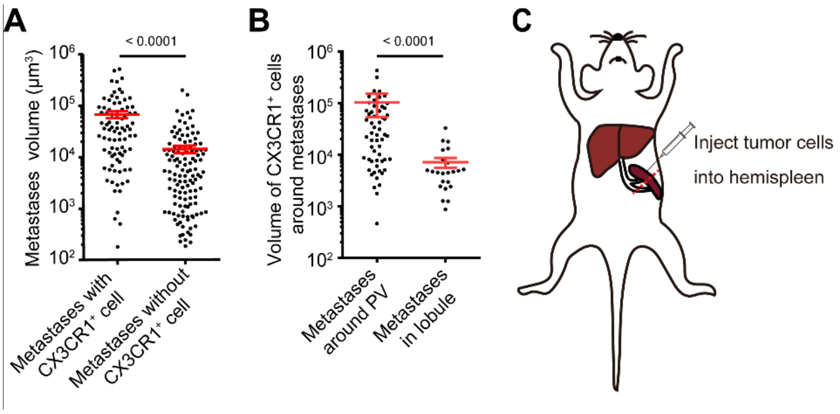

Figure S13. Quantitative analysis of the mean volume of tumor metastases and CX3CR1$^+$ cells. (A) Quantitative analysis of the mean volume of tumor metastases distributed with and without CX3CR1$^+$ cells. (B) Quantitative analysis of the volume of CX3CR1$^+$ cells around tumor metastases. (C) Schematic of tumor injection into the hemispleen.

Figure S14 M2/M1 ratio of CX3CR1$^+$ macrophages in early colorectal liver metastases

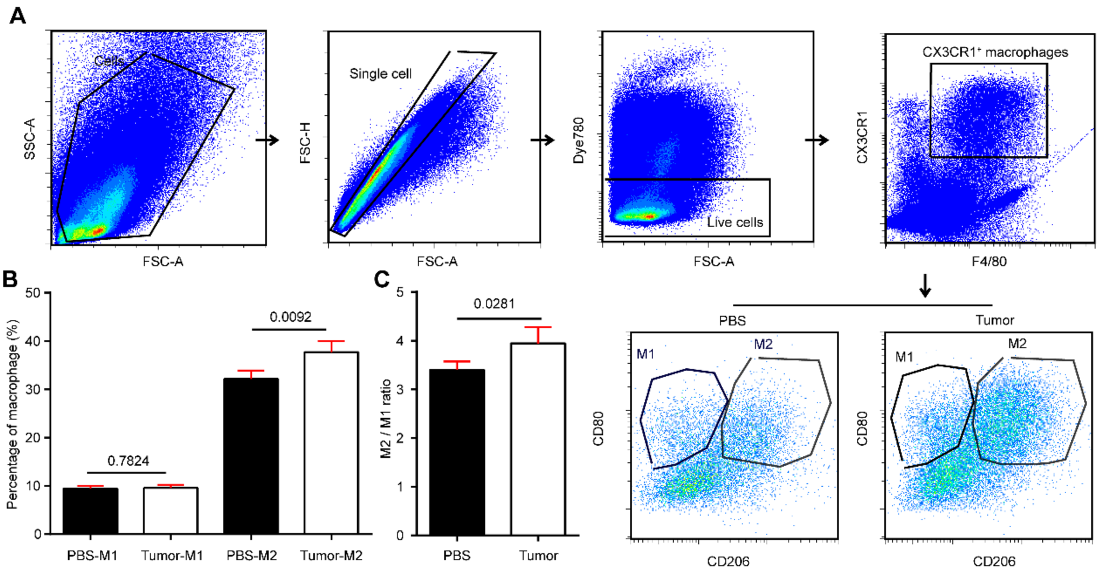

Figure S14. Flow cytometric analysis of M2/M1 ratio of CX3CR1$^+$ macrophages in early colorectal liver metastases. (A) Gating strategy for CX3CR1$^+$ macrophages in liver NPCs. (B) Average percentages of M1 and M2 cells in CX3CR1$^+$ macrophages; n = 4 mice. The error bars denote the SDs. (C) Average M2/M1 ratio in CX3CR1$^+$ cells; n = 4 mice. The error bars denote the SDs. CX3CR1$^+$ M1 cell was gated as F4/80$^+$CD206$^-$CD80$^+$, and CX3CR1$^+$ M2 cell was gated as F4/80$^+$ CD206$^+$.

Figure S15 Identification of the CCL2 knockout efficiency in MC38 cell lines

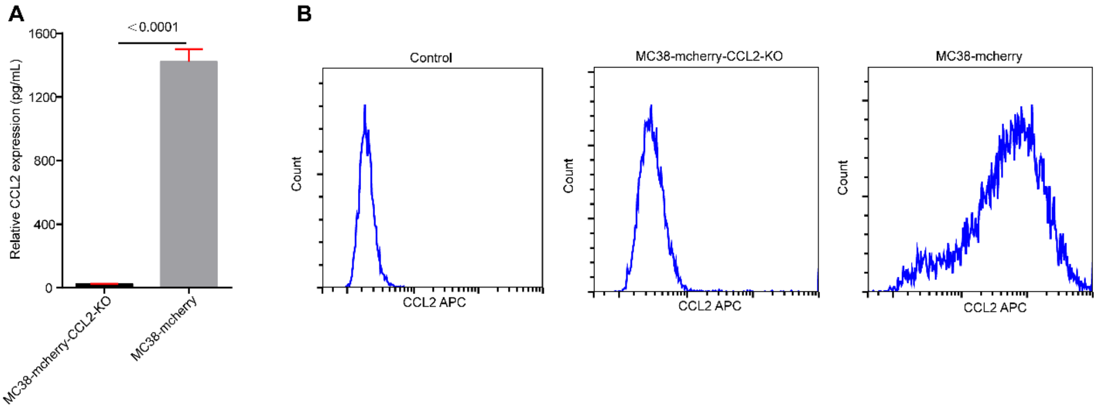

Figure S15. Identification of the CCL2 knockout efficiency in MC38 cell lines. (A) ELISA quantification of the CCL2 concentration in the cell culture supernatant of MC38-mCherry-CCL2-

KO and MC38-mCherry cell lines. (B) Flow cytometric analysis of CCL2 expression in MC38-mCherry-CCL2-KO and MC38-mCherry cell lines. MC38-mCherry cells without APC anti-mouse CCL2 labeling served as a control.

Supplementary Table 1 Parameters of the sinusoid network connectivity

	Mean	Std. deviation	Std. error of the mean
Clustering coefficient	0.038	0.006	0.002
Connected components	259	283	79
Network diameter	72	15	4
Network radius	1	0	0
Network centralization	< 0.001	< 0.001	< 0.001
Shortest paths	1.63E+08	1.40E+08	3.88E+07
Characteristic path length	29	5	1
Avg. number of neighbors	2.6718	0.1593	0.0442
Number of nodes	12399	5756	1596
Network density	< 0.001	< 0.001	< 0.001
Network heterogeneity	0.331	0.052	0.014
Isolated nodes	0	0	0
Number of self-loops	56	37	10
Multi-edge node pairs	307	171	47

Supplementary Table 2 Details of the antibodies used for immunofluorescence staining

Antibody	Catalog number	Clone number	Company	Dilution
PE-conjugated anti-mouse MHC II	107607	M5/114.15.2	BioLegend	1:200
Alexa Fluor 594-conjugated anti-mouse F4/80	123140	BM8	BioLegend	1:200
PE-conjugated anti-mouse Ly6C	128008	HK1.4	BioLegend	1:100
Rabbit anti-mouse CCR2	ab216863	EPR20844	Abcam	1:100
Alexa Fluor 647-conjugated goat anti-rabbit IgG	A21245	AB_2535813	Invitrogen	1:1000
BV421-conjugated anti-mouse MHC II	107632	M5/114/15/2	Invitrogen	1:50
BV605-conjugated anti-mouse CD45	103140	30-F11	Invitrogen	1:50
PE-conjugated anti-mouse CD86	159204	A17199A	BioLegend	1:100
Rabbit anti-mouse CK19	ab52625	EP1580Y	Abcam	1:200

Supplementary Table 3 Details of the antibodies used for flow cytometry

Antibody	Catalog number	Clone number	Company	Dilution
PerCP-Cy5.5-conjugated anti-mouse MHC II	107626	M5/114.15.2	BioLegend	1:200
BV421-conjugated anti-mouse CD11c	565452	M418	BD Pharmingen	1:200
BV605-conjugated anti-mouse CD64	139323	X54-5/7.1	BioLegend	1:100
AF647-conjugated anti-mouse CD80	104718	16-10A1	BioLegend	1:100
PE-conjugated anti-mouse CD80	104708	16-10A1	BioLegend	1:100
APC-conjugated anti-mouse CD86	558703	GL-1	BD Pharmingen	1:100
APC-conjugated anti-mouse CD40	124611	3/23	BioLegend	1:100
BV421-conjugated anti-mouse F4/80	565411	T45-2342	BD Pharmingen	1:100
BV510-conjugated anti-mouse MHC II	107636	M5/114.15.2	BioLegend	1:200
APC-conjugated anti-mouse CD206	141708	C068C2	BioLegend	1:100
APC-conjugated anti-mouse CCR2	505909	2H5	BioLegend	1:100

Supplementary Table 4 Abbreviations used in the text

**Full name**	**Abbreviation**
hepatic artery	HA
portal vein	PV
hepatic vein	HV
central vein	CV
portal triads	PTs

classic lobules	CLs
portal lobules	PLs
dendritic cells	DCs
computed tomography	CT
magnetic resonance imaging	MRI
paraformaldehyde	PFA
Akaike information criterion	AIC
See Deep Brain	SeeDB
ultimate imaging of solvent-cleared organs	uDISCO
unobstructed brain imaging cocktails and computational analysis	CUBIC
polyethylene (PEG)-associated solvent system	PEGASOS
nonparenchymal cells	NPCs
three-dimension	3D
carbon tetrachloride	CCl_4
knock-out	KO
fluorescent micro-optical sectioning tomography	fMOST

Supplementary movie legends

Supplementary movie 1. Imaging of RFP cells from LysM-RFP mouse livers subjected to liver-CUBIC and CUBIC at different depths. The Z-direction was a 50-μm maximum intensity projection. Scale bar: 200 μm.

Supplementary movie 2. Imaging of YFP cells from CD11c-YFP mouse livers subjected to liver-CUBIC and CUBIC at different depths. The Z-direction was a 50-μm maximum intensity projection. Scale bar: 200 μm.

Supplementary movie 3. Segmentation and reconstitution of the macrocirculation in a liver lobe. Imaging of an intact liver lobe and segmentation of blood vessels based on liver-CUBIC technology; related to Figure 2. The HV is shown in green, the PV is shown in red, and the hepatic lobules are shown in magenta.

Supplementary movie 4. 3D imaging of hepatic lobules and $CD11c^{+}$ cells in an intact liver lobe. The first part of the video shows the imaging of immune cells and blood vessels in a liver lobe and the segmentation of the HV and PV. The second part of the video shows the segmentation of hepatic lobules.

Supplementary movie 5. Imaging of hepatic sinusoids at different depths in a healthy liver. Ortho slice view of hepatic sinusoids in hepatic lobules. The red color represents the sinusoids in the lobular region.

Supplementary movie 6. Imaging of hepatic sinusoids at different depths in CCl_4-induced fibrosis. Ortho slice view of hepatic sinusoids in hepatic lobules. The red color represents the sinusoids in the lobular region.

Supplementary movie 7. 3D reconstruction of the sinusoid network in a healthy liver. The represented lobular sinusoids are colored, and other sinusoids are shown in white. The skeleton of the hepatic sinusoid is shown in yellow.

Supplementary movie 8. Distribution of $CD11c^+$ cells in a hepatic lobule segmented from 3D data ($1.2 \times 1.2 \times 1\ mm^3$). The HV is shown in blue, the PV is shown in red, $CD11c^+$ cells are shown in green, and $CD11c^+$ cells in the interesting hepatic lobule are shown in yellow.

Supplementary movie 9. Distribution of $CD11c^+$ cells in CCl_4-induced fibrosis hepatic lobules segmented from 3D data ($1.2 \times 1.2 \times 1\ mm^3$). The PV is shown in blue, the HV is shown in red, $CD11c^+$ cells are shown in green, and $CD11c^+$ cells in the interesting hepatic lobule are shown in yellow.

Supplementary movie 10. 3D imaging of a liver lobe with tumor metastases. Imaging of CX3CR1-EGFP cells and liver metastases in an intact liver lobe; related to Figure 7. The video shows the relationship between the locations and the distributions of tumor metastases (red), CX3CR1-GFP cells (green), and hepatic vessels (white).

Reference

1. Kubota SI, Takahashi K, Nishida J, Morishita Y, Ehata S, Tainaka K, et al. Whole-Body Profiling of Cancer Metastasis with Single-Cell Resolution. Cell Rep. 2017; 20: 236-50.
